# *Prunus* transcription factors: breeding perspectives

**DOI:** 10.3389/fpls.2015.00443

**Published:** 2015-06-12

**Authors:** Valmor J. Bianchi, Manuel Rubio, Livio Trainotti, Ignazio Verde, Claudio Bonghi, Pedro Martínez-Gómez

**Affiliations:** ^1^Department of Plant Physiology, Instituto de Biologia, Universidade Federal de PelotasPelotas-RS, Brazil; ^2^Department of Plant Breeding, Centro de Edafología y Biología Aplicada del Segura, Consejo Superior de Investigaciones CientíficasMurcia, Spain; ^3^Department of Biology, University of PaduaPadova, Italy; ^4^Consiglio per la ricerca in agricoltura e l'analisi dell'economia agraria (CRA) - Centro di ricerca per la frutticolturaRoma, Italy; ^5^Department of Agronomy, Food, Natural Resources, and Environment (DAFNAE). University of PaduaPadova, Italy

**Keywords:** *Prunus* spp., breeding, gene regulation, transcription factors, flowering time, fruit quality, abiotic stress, biotic stress

## Abstract

Many plant processes depend on differential gene expression, which is generally controlled by complex proteins called transcription factors (TFs). In peach, 1533 TFs have been identified, accounting for about 5.5% of the 27,852 protein-coding genes. These TFs are the reference for the rest of the *Prunus* species. TF studies in *Prunus* have been performed on the gene expression analysis of different agronomic traits, including control of the flowering process, fruit quality, and biotic and abiotic stress resistance. These studies, using quantitative RT-PCR, have mainly been performed in peach, and to a lesser extent in other species, including almond, apricot, black cherry, Fuji cherry, Japanese apricot, plum, and sour and sweet cherry. Other tools have also been used in TF studies, including cDNA-AFLP, LC-ESI-MS, RNA, and DNA blotting or mapping. More recently, new tools assayed include microarray and high-throughput DNA sequencing (DNA-Seq) and RNA sequencing (RNA-Seq). New functional genomics opportunities include genome resequencing and the well-known synteny among *Prunus* genomes and transcriptomes. These new functional studies should be applied in breeding programs in the development of molecular markers. With the genome sequences available, some strategies that have been used in model systems (such as SNP genotyping assays and genotyping-by-sequencing) may be applicable in the functional analysis of *Prunus* TFs as well. In addition, the knowledge of the gene functions and position in the peach reference genome of the TFs represents an additional advantage. These facts could greatly facilitate the isolation of genes via QTL (quantitative trait loci) map-based cloning in the different *Prunus* species, following the association of these TFs with the identified QTLs using the peach reference genome.

## Introduction

Transcription is a complex process in which a DNA strand provides the information for the synthesis of an RNA strand, which transfers the genetic information required for protein synthesis (Watson et al., 2014).

**Figure 1:**
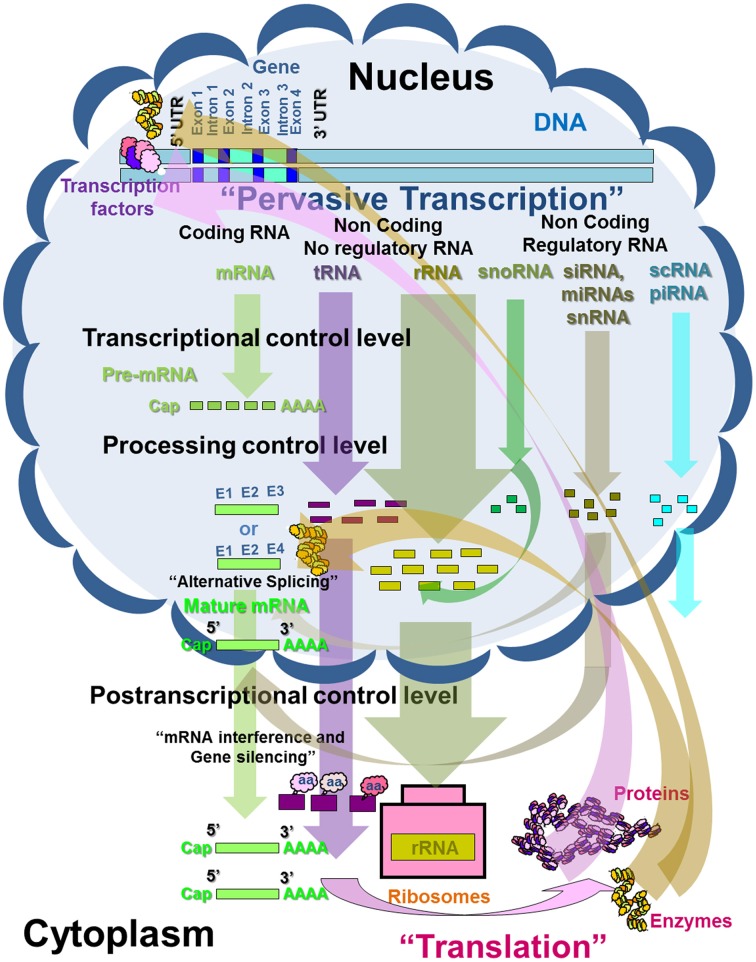
**Schematic representation of the transcription control in eukaryotes and *Prunus* (adapted from Atkins et al., 2011; Watson et al., 2014)**.

RNA molecules include coding and non-coding RNA. Protein-coding RNA is also called messenger RNA (mRNA) and makes up around 5% of the total RNA in plants. Non-coding RNA includes non-regulatory RNA and is composed of ribosomal RNA (rRNA, up to 85%) and transfer RNA (tRNA, around 15%). In addition, non-coding RNA includes regulatory RNA (less than 5%) with the group of small RNAs (sRNAs); small nuclear RNAs (snRNAs) involved in mRNA and tRNA processing; small interfering RNA (siRNA) and micro RNA involved in mRNA translation; and small cytoplasmic RNA (scRNA) and piwi-interacting RNA (piRNA), with a variable and uncertain function (Figure 1) (Atkins et al., 2011; Watson et al., 2014).

The coding and noncoding-regulatory RNAs are the main molecules involved in the transcription process. This molecule occurs in a highly selective process in which individual genes (monocistronic transcription) are transcribed only when their products, the respective proteins, are required for a cell, a group of cells, or an organ, as a result of spatial and temporal plant growth and development control. The enzymes responsible for transcription in living organisms, including plants, are called RNA polymerases (RNAPs). Plants contain the following four distinct RNA polymerase enzymes, each responsible for synthesizing a different RNA molecule: RNA polymerase I (larger rRNAs); RNA polymerase II (pre mRNAs, snoRNAs -small nucleolar RNAs-, snRNAs, miRNAs); RNA polymerase III (scRNAs, tRNAs, smaller rRNAs); and RNA polymerase IV (siRNAs), which is specific to plants (Kornberg, 2007; Krishnamurthy and Hampsey, 2008). The point on the DNA to which an RNA polymerase enzyme binds prior to initiating transcription is called the promoter. Yet this enzyme is not capable of recognizing promoter regions and requires the help of a large variety of accessory proteins called transcription factors (TFs) (Karp, 2008; Krishnamurthy and Hampsey, 2008).

TFs are proteins that bind a specific DNA sequence and thereby regulate the expression of target genes (Krishnamurthy and Hampsey, 2008). TF/RNAP interaction is thus necessary to form what is also known as the pre-initiation complex to start the transcription process. The same TFs can be involved in the transcription process as co-activators, acting in chromatin remodeling, histone acetylation and nucleic acid methylation, thus up- and down-regulating gene expression (Kornberg, 2007; Watson et al., 2014).

**Figure 2:**
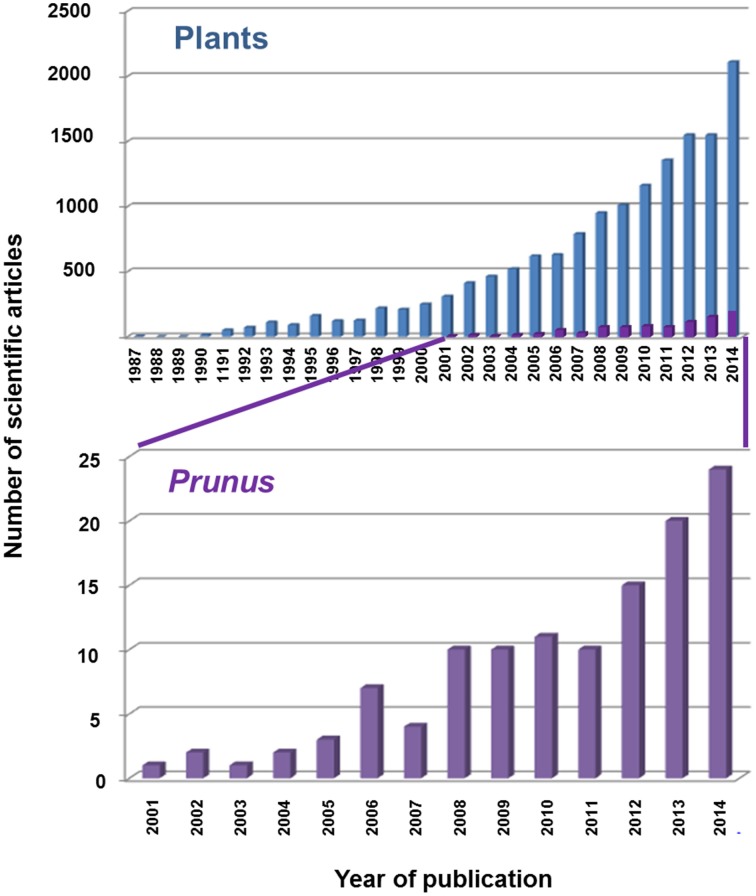
**Number of scientific articles related to *Prunus* transcription factors published in the WOK database (Web of Knowledge, http://apps.webofknowledge.com)**.

TFs are crucial for the action of the RNAPs, but they have mainly been studied in the case of mRNAs and RNA polymerase II. All major processes of life depend on differential gene expression, which is generally controlled by these TFs (Kornberg, 2007; Karp, 2008; Atkins et al., 2011). The first TFs were described in plants in the 1980s, yet only around 1400 scientific articles about TFs had been published by the year 2000. In the last 14 years, however, with the newly available strategies and tools for molecular studies, TF studies have increased exponentially. Indeed, more than 13,000 articles have been published in plants during this time period. In the case of *Prunus* species, TF studies have also increased exponentially since 2001. This is indicative of how much remains to be done in order to discover and better understand the real function of TFs and how they influence the main characteristics of agronomical importance in *Prunus* spp. (Figure 2).

Two main plant TF databases are currently available online: the Plant Transcription Factor Database v3.0 (PlnTFDB) (http://plntfdb.bio.uni-potsdam.de/v3.0/) of the University of Potsdam (Germany) (Pérez-Rodríguez et al., 2009) and the Plant Transcription Factor Database v3.0 (PlantTFDB) (http://planttfdb.cbi.pku.edu.cn/) of the Centre for Bioinformatics of Peking University (China) (Jin et al., 2014). In general, the information and terminology is similar in both databases, although there are some discrepancies, mainly involving the nomenclature of the different TF families. Information regarding *Prunus* TFs, however, is only available in the PlantTFDB database. According to this database, TFs encoded by the different plant genomes can be classified into 57 major multigene families, including 123,497 different TFs identified (Table 1). The largest families are the basic helix-loop-helix (bHLH) family, the ERF (mTERF) family, the MYB family and the NAC family, all of which have more than 8000 members in this database. The members/genes of these four super-families of TFs are involved in a wide range of biological processes, like the control of mtDNA replication, embryo development, flower and fruit development, fruit dehiscence, meristem determinacy, cell proliferation and differentiation, among others (Littlewood and Evan, 1995; Souer et al., 1996; Roberti et al., 2009) (Table 1).

**Table 1:** **Transcription factor (TF) families identified in plants and peach available in the PlantTFDB database (http://planttfdb.cbi.pku.edu.cn/)**.

**Family**	**Brief description of function**	**Number of TFs described in**		**First reference**
		**Plants**	**Peach**	
AP2 (EREBP)	Regulate developmental processes	1766	19	Ohme-Takagi and Shinshi, 1995
ARF	Regulate the expression of auxins	1914	17	Ulmasov et al., 1997
ARR-B	Signal transduction for propagation	914	12	D'Agostino and Kieber, 1999
B3 (ABI3VP1)	Seed dormancy/DNA binding	4051	66	Suzuki et al., 1997
BBR/BPC	Control of ovule identity	492	4	Santi et al., 2003
BES1	Regulate BR-induced genes	651	9	Yin et al., 2005
bHLH	Essential developmental processes	11,428	133	Littlewood and Evan, 1995
bZIP	Pathogen defense, light and stress	6258	50	Landschulz et al., 1988
CAMTA (TIG)	Regulate CBF2 expression	518	4	Bouché et al., 2002
C2H2 (ZF)	Protein-protein interactions	7336	80	Takatsuji, 1999
C3H	Regulate embryogenesis	4019	46	Li and Thomas, 1998
CO-like	Flowering induction	854	9	Lagercrantz and Axelsson, 2000
CPP	Regulate leghemoglobin	594	6	Cvitanich et al., 2000
DBB	Photomorphogenis of hypocotyl	764	6	Kumagai et al., 2008
Dof	Plant growth and development	2312	26	Yanagisawa, 1997
E2F-DP	Control of cell cycle	692	6	Zheng et al., 1999
EIL	Ethylene signaling	531	4	Solano et al., 1998
ERF (mTERF)	Control of mtDNA replication	8688	107	Roberti et al., 2009
FAR1	Modulate phyA-signaling homeost.	2542	78	Hudson et al., 1999
G2-like	Establishment of polarity	3935	36	Eshed et al., 2001
GATA	Light-responsive transcription	2229	22	Teakle et al., 2002
GeBP	Leaf cell fate and cytokinin response	683	8	Curaba et al., 2003
GRAS	Root and shoot development	3915	49	Richards et al., 2000
GRF	Regulation of cell expansion	752	10	Kim et al., 2003
HB-other	Maintain Homeodomiun functionalit.	987	7	Ariel et al., 2007
HB-PHD	Regulate domain of PHDf proteins	160	2	Halbach et al., 2000
HD-ZIP	Dimmers to recognize DNA	3436	33	Ariel et al., 2007
HRT-like	Developmental and phytohormone	95	1	Raventós et al., 1998
HSF	Regulate heat shock expression	1833	21	Fujita et al., 1989
LBD	Recognize the cis-element GCGGCG	2779	42	Husbands et al., 2007
LFY	Flower development	100	1	Parcy et al., 1998
LSD	Regulate plant cell death	402	4	Dietrich et al., 1997
M (MADS-BOX)	Floral meristem and organ identity	2978	52	Shore and Sharrocks, 1995
MIKC	Floral organ identity determination	2864	28	Nam et al., 2003
MYB	Proliferation and differentiation of cell	8746	121	Stracke et al., 2001
MYB-related	DNA-binding	6410	55	Kirik and Baumlein, 1996
NAC	Plant development and stress response	8133	115	Souer et al., 1996
NF-X1	Protein interactions under stress	146	2	Lisso et al., 2006
NF-YA	Control flower timer / drought stress	943	7	Siefers et al., 2009
NF-YB	Motifs of H2B histone/ drought stress	1334	14	Siefers et al., 2009
NF-YC	Motifs of H2A histone/ drought stress	1018	9	Siefers et al., 2009
Nin-Like	Nodule primordial initiation	1002	8	Schauser et al., 1999
NZZ/SPL	Control male and female sporogenesis	45	2	Schiefthaler et al., 1999
RAV	Ethylene and brassinosteroid response	289	5	Nole-Wilson and Krizek, 2000
S1Fa-like	Tissue-specific negative elements	158	5	Zhou et al., 1995
SAP	Flower and ovule development	63	1	Byzova et al., 1999
SBP	Flower and fruit development	1675	17	Klein et al., 1996
SRS	Control GA responses	506	6	Fridborg et al., 1999
STAT	Morphogenesis and cell regulation	84	1	Yamada et al., 2008
TALE	Meristem morphogenesis	1797	22	Ariel et al., 2007
TCP	Floral zygomorphy, apical dominance	1704	19	Cubas et al., 1999
Trihelix (GT)	Fruit and seed development	2599	33	Smalle et al., 1998
VOZ	Plant development	227	3	Mitsuda et al., 2004
Whirly	Basal and specific defense responses	233	2	Desveaux et al., 2005
WOX	Promotion of cell division activity	937	10	Ariel et al., 2007
YABBY	Abaxial identity in apical and flower	725	7	Golz and Hudson, 1999
WRKY	Biotic and abiotic stress responses	5936	61	Eulgem et al., 2000
Zf-HD	Expression pattern of the C4 PEPCase	1066	10	Windhövel et al., 2001

The purpose of this study was to summarize the information available from the TF studies in *Prunus* spp., based on a review of the bibliography. The availability of the peach genome sequence (Verde et al., 2013) made it possible to make an inventory of peach TFs at the whole genome level. This paper also includes a discussion of the main implications of this information for the breeding and development of marker-assisted selection strategies, with particular focus on characteristics of direct agronomical interest.

## Transcription factors identified in *Prunus*

The *Prunus* genus inside the *Rosaceae* family and the *Rosales* order is widely grown around the world and includes about 230 species, many of which produce edible fruits and seeds of economic interest (Potter, 2012). Inside this genus, peach [*Prunus persica* (L.) Batsch] presents several physiological and molecular advantages, including self-compatibility, a short juvenile phase and a small genome size (227.3 Mb). These characteristics make peach a suitable model species within the *Prunus* genus and even within the *Rosaceae* family (Arús et al., 2012). The complete peach genome sequence (Peach v1.0) was recently published (Verde et al., 2013) and is now the reference genome in these species. Within this genome, 1529 TFs have been identified to date in the PlantTFDB database, accounting for about 5.3% of the 27,864 protein-coding genes identified in peach. This proportion is similar to that described in *Arabidopsis*, where Riechmann (2006) estimated that of the 26,000 protein-coding genes, 6.4% were TFs. Among the TF families encoded by the different plant genomes, around 30 families have more than 1000 member genes identified in the PlantTFDB database. With respect to the peach genome, only four families of these TFs [bHLH; ERF (mTERF); MYB; and NAC] have more than 100 identified members, and just 10 of these TFs have 50 or more member genes per family (Table 1). As regards the family size comparison, members of FAR1 are more abundant than members in the *Arabidopsis* and poplar genomes, while ARF, SBP, ARR-B, CO-like, NF-YA, SRS, BBR/BPC, and LSD families are smaller (Table 1; Supplementary Material, Table S1).

The information about TFs contained in the PlantTFDB database was checked in the GDR database (www.rosecae.org), revealing a wide distribution of the 1529 TFs identified in peach, with a higher number of TFs (transcripts) on pseudomolecule 1 (312 TFs) (Table 2; Supplementary Material, Table S2).

**Table 2:** **Number and distribution of Transcription Factors (TFs) in each pseudomolecule (Scaffold1-8) of the Peach v1.0 genome sequence**.

	**Scaffold 1**	**Scaffold 2**	**Scaffold 3**	**Scaffold 4**	**Scaffold 5**	**Scaffold 6**	**Scaffold 7**	**Scaffold 8**	**Others**
Total transcription factors	323 (325)^a^	181 (194)	159 (173)	167 (158)	174	198 (200)	188 (180)	124 (125)	19^b^ (4)
TFs of sequences without isoforms	312	176	155	162	170	195	184	118	
Repeated TF sequences^c^	9	2	4	3	4	3	4	6	
Sequences partially/completely overlapping	2	3	1	2	–	–	–	–	

a *The putative number of Transcription Factors for each pseudomolecule in the updated peach version according to the assembly refinements described in Verde et al. (2013, 2015) is reported in brackets*.

b *Fifteen of these TFs are included within the 8 psudomolecules since the unmapped scaffolds where they are located were mapped according to the assembly refinements described in Verde et al. (2013) and now included within the 8 pseudomolecules in v2.0 assembly (Verde et al., 2015)*.

c *Mostly alternative transcripts*.

In fruit trees, including *Prunus*, understanding how morphological and phenological traits (flowering timing, bud dormancy, bud and fruit development, cultivar acclimation, chilling requirement, among others) behave in different and changing environments is very important in the search to identify genotypes with better fruit quality, productivity and growth potential to be used in breeding programs. Accordingly, TF studies in *Prunus* have been performed at the gene expression level for several agronomic traits, such as control of the flowering process, tree shape, fruit quality, and drought and disease resistance, which are found not only in peach, but also in other *Prunus* species. These TF studies have mainly been performed in peach (Table 3) and to a lesser extent in other species, including almond [*P. amygdalus* (Batsch) syn. *P. dulcis* (Miller) Webb], apricot (*P. armeniaca* L.), black cherry (*P. serotina* Ehrh), fuji cherry (*P. incisa* Thunb.), Japanese apricot (*P. mume* Sieb. Et Zucc.), Japanese plum (*P. salicina* Lindl), and sour (*P. cerasus* L.) and sweet (*P. avium* L.) cherry (Table 4). TF analyses have mainly been performed using quantitative RT-PCR to amplify the known sequences of these TFs. Other tools assayed include cDNA-AFLP, LC-ESI-MS, and RNA and DNA blotting or mapping. More recently, new tools assayed include microarray and high-throughput DNA (DNA-Seq) and RNA (RNA-Seq) sequencing (Tables 3, 4).

**Table 3:** **Transcription factors (TFs) assayed in peach in the study of different agronomic traits**.

**Agronomic traits**		**Transcription factor family**	**Analytic tool**	**Reference**
Fruit quality	Fruit storage	AP2/ERF (CBF1,5,6)	RT-PCR	Liang et al., 2013
	Ripening time	NAC	Fine mapping	Pirona et al., 2013
	Ripening process	AP2/ERF, SBP(CNR), bZIP	RT-PCR	Lovisetto et al., 2013
	Fruit ripening	bZIP, AP2/ERF, MADS-BOX	RT-PCR; over express.	Tadiello et al., 2009; Soto et al., 2012
	Aroma development	AP2/ERF, NAC, ARF(AUX/IAA)	qtr.-PCR	Sánchez et al., 2013
	Flavonoid biosynthesis	bHLH, MYB, NAC, SPL	RT-PCR; over-espression; VIGS	Ravaglia et al., 2013; Rahim et al., 2014; Zhou et al., 2015
	Split-pit formation	MADS-BOX	RT-PCR	Tani et al., 2007
	Split-pit formation	MADS-BOX, bHLH	RT-PCR	Tani et al., 2011
	Stone formation	MADS-BOX (SHP, STK), NAC (NTS)	RT-PCR	Dardick et al., 2010
	Cold acclimation	AP2/ERF, HSF (MYRC), MYB-R	RT-PCR	Tittarelli et al., 2009
	Fruit ripening	LIM	RT-PCR, Microarray	Ziosi et al., 2009
	Fruit ripening	HD-ZIP, ERF, ARF(AUX/IAA), EIL	RT-PCR, Microarray	Ziliotto et al., 2008
	Trichome formation	MYB	Mapping, RT-PCR	Vendramin et al., 2014
	Bud dormancy	MADS-BOX (DAM6)	RT-PCR	Leida et al., 2012
Flowering time	Bud dormancy	MADS-BOX (DAM4,5,6)	RT-PCR	Leida et al., 2010
	Bud dormancy	AP2	RT-PCR	Wisnieski et al., 2011
	Chilling requirement	MADS-BOX (DAM5/6)	RT-PCR	Jiménez et al., 2010
	Terminal bud formation	MADS-BOX (MIKC-DAM)	Phylogenetic analyses	Jiménez et al., 2009
	Terminal bud formation	MADS-BOX (MIKC-DAM1,2,3,4,5,6)	Mapping, RT-PCR	Bielenberg et al., 2008
	Flower development	MADS-BOX	RT-PCR, Blotting	Xu et al., 2008
	Flower development	MADS-BOX1/10	Mapping, RT-PCR	Zhang et al., 2008
	Flower development	MADS-BOX	RT-PCR	Martin et al., 2006
	Flower development	MADS-BOX (MIKC)	RT-PCR	Yamane et al., 2011
	Flower development	LFY	RT-PCR	An et al., 2012
	Bud dormancy	MADS-BOX	RNA-Seq	Wells et al., 2015
Plant Growth	Circadian cycle	AP2/ERF (CBF/DREB2)	RT-PCR	Artlip et al., 2013
	Nitrogen metabolism	DOF	DEG	Wang et al., 2012
	Anthocyanin biosynthesis	MYB	RT-PCR	Zhou et al., 2013
	Anther development	bHLH, PHD	RT-PCR	Rios et al., 2013
	Stem development	TALE (KNOX/KNOPE1)	Mapping, RT-PCR	Testone et al., 2012
	Sugar translocation	TALE (KNOX/KNOPE3)	RT-PCR	Testone et al., 2009
	Fruit/seed development	ARF (Aux/IAA), ERF, GRAS (DELLA)	RT-PCR	Ruiz et al., 2013
	Response to low temper.	AP2/ERF (CBF/DREB), MYB, MYC	RT-PCR	Bassett et al., 2009
	Floral organ formation	MYB, AP2	RT-PCR, RLM-RACE	Gao et al., 2012a
	Leaf coloration	MYB	RNA-Seq, RT-PCR	Zhou et al., 2014
Drought resistance	Adaptation to drought	NF-YA	RT-PCR	Eldem et al., 2012
Disease resistance	Virus resistance	bHLH (MYC)	RNA-Seq	Rubio et al., 2015
	Bacteria resistance	AP2/ERF	RT-PCR	Sherif et al., 2013
	Bacteria resistance	ERF, MYC	RT-PCR	Sherif et al., 2012
	Bacteria resistance	ERF, MYB, bHLH, WRKY	RNA-Seq, RT-PCR	Socquet-Juglard et al., 2013
	Fungal resistance	CTF1α and 1β AP-l/CRE1 NIT2	sqRT-PCR	Lee et al., 2010
	Plant/Virus interaction	eEF1A	RT-PCR	Dubé et al., 2009
	Response to biotic stress	CHL P	RT-PCR	Giannino et al., 2004
	Virus resistance	Translation initiation factor (eIF4E)	Mapping	Lalli et al., 2005
	Fungi infection	TALE (KNOX/KNOPE1)	RT-PCR	Testone et al., 2008
	Nematode resistance	WRKY	Positional cloning	Claverie et al., 2011

**Table 4:** **Transcription factors (TFs) assayed in almond, apricot, black cherry, sweet cherry, Japanese apricot, and Plum in the study of different agronomic traits**.

**Species**	**Agronomic traits**		**Transcription factor family**	**Analytic tool**	**Reference**
Almond	Abiotic stresses	Drought resistance	bHLH, MYB	cDNA-AFLP	Alimohammadi et al., 2013
		Cold acclimation	AP2/EFR (PdCBF1,2)	RT-PCR	Barros et al., 2012a
		Cold acclimation	AP2/EFR (PdCBF1,2),	RT-PCR	Barros et al., 2012b
		Cold acclimation	AP2/EFR (CBF/DREB1)	RNA-Seq, RT-PCR	Mousavi et al., 2014
	Floral development	Ovule emergence	MADS-BOX (PdMADS-BOX1,3)	RT-PCR	Barros et al., 2012b
		Flowering time	MADS-BOX, LFY	Mapping	Silva et al., 2005
	Meristem development	Shoot meristem formation	TALE (KNOTTED)	RT-PCR	Santos et al., 2012
Apricot	Chilling requirement	Bud dormancy	MADS-BOX	RT-PCR	Trainin et al., 2013
	Disease resistance	Virus resistance	TRAF	DNA-Seq	Zuriaga et al., 2013
	Fruit development	Fruit ripening	bZIP, MYB-type	RT-PCR	Manganaris et al., 2011
	Cross-pollination	Pollen and pistil interactions	MYB	LC-ESI-MS	Feng et al., 2006
Black cherry	Floral development	Flower morphogenesis	MADS-BOX	RT-PCR	Liu et al., 2010
Fuji cherry	Plant growth	Somatic embryogenesis	E2F-DP, ARF (ABP)	RT-PCR	Ben Mahmoud et al., 2013
Japan. apricot	Plant development	Plant development	AP2/ERF	RT-PCR	Du et al., 2013
	Abiotic stress	Response to low temperatures	AP2/EFR (PmCBFa,b)	RT-PCR	Zhang et al., 2013
		Response to low temperatures	AP2/EFR (PmCBFb,c)	RT-PCR	Guo et al., 2014
	Fruit development	Fruit ripening process	NAC	RT-PCR	Mita et al., 2006
	Flower development	Pistil development	ARF2	RT-PCR	Gao et al., 2012b
		Bud endodormancy	MADS-BOX (MYKC)	SSH/MOS	Yamane et al., 2008
Japanese Plum	Fruit development	Fruit ripening process	AP2/EREBP	RT-PCR	El-Sharkawy et al., 2007
		Fruit ripening process	AP2/EREBP	RT-PCR	El-Sharkawy et al., 2009
Sour cherry	Abiotic stress	Freezing tolerance	AP2/EFR (CBF1)	Heter. expression	Owens et al., 2002
Sweet cherry	Abiotic stress	Freezing tolerance	AP2/EFR (CBF/DREB1)	PCR, RNA blotting	Kitashiba et al., 2002
	Seed germination	Primary seed dormancy	B3 (ABI3/VP1)	RT-PCR	Stephen et al., 2004
	Fruit quality	Fruit skin and flesh colors	MYB	Mapping	Sooriyapathirana et al., 2010
		Flavonoid biosynthesis	bHLH (MYB)	RT-PCR	Shen et al., 2014
		Fruit development	AP2, ERF, HB-ZIP, MYB, NAC	RNA-Seq	Alkio et al., 2014
	Floral development	Flowering time	MADS-BOX	RT-PCR	Wang et al., 2013

## Flowering date control

Late flowering is an important agronomic trait for avoiding spring frost in *Prunus* species, particularly in the case of the earlier flowering species such as almond. Furthermore, the development of cultivars with early flowering has made *Prunus* species production a reality in subtropical areas. More knowledge about the factors involved (TFs) in the control of dormancy and flowering date can help in the development of new *Prunus* genotypes with either later flowering dates and higher chilling requirements to break dormancy to avoid frost or earlier flowering dates and lower chilling requirements to be grown in subtropical areas (peach and Japanese plum) for early production (Wells et al., 2015).

In the case of *Prunus* and other woody plants of the *Rosaceae* family, such as apple and pear, dormancy is a mechanism that allows the plants to withstand low temperatures and acclimate to winter conditions. There is usually a relationship between flowering date, bud dormancy, and chilling and heat requirements (Sánchez-Pérez et al., 2012, 2014). In the case of peach, several members of the MADS-BOX TF family (*MIKC-DAM 1, 2, 3, 4, 5*, and *6*) are differentially expressed and have been associated with the control of genes responsible for arresting meristem development, for terminal bud formation and for bud dormancy (Bielenberg et al., 2008; Jiménez et al., 2009). In *Arabidopsis*, MADS-BOX TFs have been identified as being involved in floral organ identity and in the control of petal, stamen, and carpel development (Parenicová et al., 2003).

In peach, a group of *DAM* (*dormancy-associated*) *SVP*-like (*Short Vegetative Phase*) MADS-BOX TFs located in the *evergrowing* (*EVG*) region has been described as being responsible for the absence of vegetative endodormancy (Li et al., 2009; Jiménez et al., 2010). This MADS-BOX domain (from the founding *MCM1*, *AGAMOUS*, *DEFICIENS*, and *SRF* TFs) is a conserved DNA-binding region present in a variety of TFs representing a large multigene family in plants. In the peach genome, 79 MADS-BOX TFs have been described, and their annotation has been manually curated (Verde et al., 2013; Wells et al., 2015). Many of the genes of the MADS-BOX family are involved in different steps of flower development, including flowering time determination (Riechmann and Meyerowitz, 1998); bud dormancy (Leida et al., 2010, 2012; Zhong et al., 2013); terminal bud formation (Bielenberg et al., 2008; Jiménez et al., 2009); and flower development (Martin et al., 2006; Xu et al., 2008; Zhang et al., 2008). Yamane et al. (2011) analyzed the expression of *PpDAM5* and *PpDAM6* during flower bud development in peach cultivars with different chilling requirements, finding that both genes are up-regulated during flower organ differentiation and then down-regulated during flower organ enlargement. Similar patterns of expression for *PmDAM5* and *PmDAM6* genes were observed in *P. mume* by Zhong et al. (2013), suggesting that these genes might contribute significantly to terminal bud set and dormancy induction and that their transcript levels could thus provide some sort of measurement of the specific chilling requirements for dormancy release.

It has recently been reported that the expression of *DAM 5* and *6* peach genes can be controlled by chromatin remodeling and modification factors [e.g., a putative *SWI3C*-like element of the *SWITCH/SUCROSE NONFERMENTING* (*SWI/SNF*) remodeling complex (ppa001566m); an *HDA2*-like histone deacetylase (ppa006590m); and a *HAM2*-like histone acetyltransferase (ppa005747m)] that are co-localized in the same quantitative trait locus (QTL) (Romeu et al., 2014). It is worthy to note that *DAM 6* from peach is regulated at the chromatin level by demethylation of *H3K4*, trimethylation of *H3K27* and acetylation of *H3* following chill accumulation (Leida et al., 2012).

In Japanese apricot, different ARF-related TFs appear to play an important role during the four stages of seasonal bud dormancy by regulating (both as inducers and repressors) the transcription of auxin-related genes and, thus, the responsiveness to auxin. The interaction between ethylene, ABA, and JA in the transition among the different dormancy phases is worthy of note (Zhong et al., 2013). In particular, Zhong et al. (2013) reported that the *JA Carboxy 1 Methyltransferase*, *EFR1* and *ERF5* genes were down-regulated in endodormancy compared with the paradormancy stage, suggesting a strong interaction between JA and ethylene in the establishment of dormancy. On the contrary, two ABA-related genes (ppa006696m and ppa008716m) were up-regulated in endodormancy and presented lower expression levels in the paradormancy stage.

Another class of TFs belonging to the CBF family has been well-documented as being related to cold response and acclimation in peach, almond, apricot, cherry, and Japanese apricot (Kitashiba et al., 2002; Owens et al., 2002; Tittarelli et al., 2009; Li et al., 2009; Barros et al., 2012a,b; Trainin et al., 2013; Zhang et al., 2013; Guo et al., 2014). CBF proteins belong to the *CBF/DRE* binding (*DREB*) sub-family of the Apetala2-ethylene responsive factor (AP2/ERF) (Nakano et al., 2006). AP2/ERF or AP2/EREBD (Ethylene Responsive Element Binding Factor) is a multigene superfamily of TFs that act under different growth and developmental mechanisms used by plants to respond to several biological processes and to several types of biotic and abiotic stresses. This TF family is large but unique to plants, and the identity of sequences among different *AP2/ERF* genes has been estimated to be as low as 13% (Riechmann and Meyerowitz, 1998; Sakuma et al., 2002). This TF family is divided into three subfamilies: the AP2 family proteins that contain two repeated AP2/ERF domains; the EREBP genes with a single AP2/ERF domain (Shigyo et al., 2006); and the RAV family proteins that contain a B3 domain, which is a DNA-binding domain conserved in other plant-specific TFs, in addition to the single AP2/ERF domain (Nakano et al., 2006). Proteins of the AP2/ERF family have been shown to participate in the regulation of developmental processes, like flower development, spikelet meristem determinacy, leaf epidermal cell identity, and embryo development.

Zhebentyayeva et al. (2014) developed a comprehensive program to identify genetic pathways and potential epigenetic mechanisms involved in the control of chilling requirement and flowering time in peach. In almond, integrating genomic and transcriptomic approaches, Silva et al. (2005) described several QTLs (Quantitative Trait Loci) linked to flowering time in an interspecific **F_2_** almond × peach progeny using a Candidate Gene approach (CG) including LFY and MADS-BOX TFs. More recently, two *C-repeated binding factor* genes in almond (*PdCBF1* and *PdCBF2*) were analyzed in flower buds and shoot internodes, showing that *PdCBF2* increased in transcript abundance during cold acclimation, while *PdCBF1* was expressed during the summer. Similarly, in *P. mume*, Guo et al. (2014) found that the *PmCBFa*, *PmCBFb*, and *PmCBFc* genes were cold induced, and the mRNA content was higher in the plants after 168 h of low temperature exposure than at 0 h. However, the mRNA content of *PmCBFa* and *PmCBFb* was higher than that of *PmCBFc*, especially after 168 h, suggesting fewer transcripts of this gene in the flower buds of *P. mume* in late winter. These results were attributed to the great variation among *CBF* genes, which can explain the variation in cold tolerance among *P. mume* populations. Interestingly, *CBF*-specific *CTR/DRE cis* elements in promoters of peach *PpDAM 5* and *PpDAM 6* genes were also found, suggesting their association with a *CBF-regulon* (Barros et al., 2012b).

In addition to the findings described above, *TERMINAL FLOWER1* (*TFL1*) and *FLOWERING LOCUS T* (*FT*) have been identified as being key regulators of flowering time and inflorescence development, but with antagonistic functions (Sánchez-Pérez et al., 2014). In black cherry (*Prunus serotina* Ehrh.), Wang and Pijut (2013) cloned two *TFL1* homologous genes that presented high expression levels in shoot tips and vegetative buds, acting as repressors of floral genes and in the maintenance of vegetative growth. Furthermore, it has been suggested that *TFL1* interacts with the bZIP transcription factor FD, repressing the transcription of the FD-dependent genes *AP1* and *AG*, while *FT* has an activation effect under *AP1* and *AG*. Nevertheless, these authors observed that *FT* activity was more important in the timing of flowering than *TFL1*, suggesting that *FT* and *TFL1* have opposite functions in regulating flowering time.

## Fruit and seed development

One of the main objectives of all *Prunus* breeding programs has traditionally been to obtain new genotypes with improved fruit quality according to consumer demand production costs and processes and distribution logistics. Fruit quality involves an important group of traits that determine the success of a new cultivar, such as aroma, solid soluble content (SSC), titratable acidity, health attributes, and both skin and flesh color, among other characteristics (Infante et al., 2011). The study of the implications of the different TF families in the processes related to fruit quality in the different *Prunus* species provides new opportunities for the marker-assisted breeding of genotypes with more extensive maturity date and the potential to preserve fruit quality after harvesting. Other opportunities include the identification of *Prunus* rootstocks whose endocarp shows less physical resistance to cracking by natural seed power during the germination process, which is a desirable characteristic.

Phenolic compounds are the precursors of anthocyanins, flavones and proanthocyanidin biosynthesis in the flavonoid pathway (D'Archivio et al., 2007), and these compounds also play a central role as determinants of fruit quality. The most important phenolic compounds are the antioxidant components in fresh fruit (Vinson et al., 2001). The accumulation of these compounds in fruit provides essential cultivar differentiation for consumers and represents an important factor for marketability (Andreotti et al., 2008). TFs of distinct families have been identified as regulating the transcription control of the flavonoid pathway. In this process, *R2R3*-MYB and basic Helix-Loop-Helix (bHLH) TFs form a complex with *WD40* proteins (termed the *MBW* complex) to activate the anthocyanin and proanthocyanidin biosynthetic genes (reviewed in Petroni and Tonelli, 2011). The *MBW* complex usually regulates groups of flavonoid biosynthetic genes, and this regulation is via specific binding to motifs in the promoters of the pathway genes (Hartmann et al., 2005). In apple, Espley et al. (2007) have demonstrated that the efficient induction of anthocyanin production during ripening depends upon the co-expression of MYB TFs (*MdMYB10*) and two bHLH TFs (*MdbHLH3* and *MdbHLH33*). Similarly in peach, the anthocyanin production occurring at ripening, mainly in the peel and in the mesocarp around the stone, is regulated by the coordinated action of *MYB10-like* and bHLH TFs (Rahim et al., 2014). Three highly similar *MYB10-like* genes (named *MYB10.1*, *2*, and *3*) form a small cluster on chromosome 3 and are closely associated with *Ag* (*anther color*), a trait responsible for pigment accumulation in anthers. Transactivation experiments identified PpMYB10.1 and PpbHLH3 as the best partners for the induction of anthocyanin production both in tobacco leaves and the peach mesocarp, thus indicating that the corresponding genes are good targets for genetic improvements (Rahim et al., 2014). Moreover, ppa018744, named MYB10.4, was associated with leaf red coloration in peach (Zhou et al., 2014). Furthermore, it must be noted that a major QTL for skin and flesh color has been mapped in the syntenic region of sweet cherry (Sooriyapathirana et al., 2010).

In peach (and other *Prunus* species), color formation due to anthocyanin accumulation is also important in flower petals. Pigment accumulation is also regulated by an MYB TF in the petals, although this MYB TF belongs to a different group of MYB10s (Uematsu et al., 2014). Nevertheless, other *MYB10-like* genes (ppa024617m and ppa010069m) that remain uncharacterized might be important for anthocyanin accumulation either in petals or aging leaves (Rahim et al., 2014). Moreover, ppa018744, named MYB10.4, was associated with leaf red coloration in peach (Zhou et al., 2014). This fact reveals the complexity of the regulation of anthocyanin synthesis, but at the same time it adds more possibilities for the genetic manipulation of this process.

In sweet cherry (*Prunus avium* L.), Shen et al. (2014) identified the *PacMYBA* gene, an MYB TF, that was associated with anthocyanin accumulation and that interacted with bHLH TFs to regulate the expression of anthocyanin pathway genes. Although the *PacMYBA* gene was expressed in several organs and tissues, *PacMYBA* expression was greatest in the skin of mature fruits and appeared to be directly up-regulated by ABA production. These results indicate that ABA production and *PacMYBA* expression work together to control anthocyanin synthesis in sweet cherry.

It has been claimed that the MYB TFs play an important role in other plant growth and developmental processes. Vendramin et al. (2014) characterized the gene ppa023142m (*PpeMYB25*) that encodes an MYB TF, which acts as a positive regulator in trichome formation and is responsible for the fuzzy skin trait in peach. These authors identified an insertion of a Ty1-*copia* retrotransposon within the *PpeMYB25* gene that disrupted the gene, leading to a recessive loss-of-function mutation underlying the nectarine phenotype. The involvement of MYB TFs in the regulation of epidermal cell differentiation and fruit development was also suggested by Alkio et al. (2014). In analysing the exocarp-specific transcripts of sweet cherry fruits, these authors identified the *R2R3-MYB Pa_22147* gene, which is also related to anthocyanin biosynthesis. Another three genes (*Pa_08841*, *Pa_02691*, and *Pa_19618*) related to ERF TFs were associated with the regulation of cutin and wax deposition in the exocarp. In addition to these results, the exocarp-specific *Pa_05584* gene, an HD-ZIP-related TF, showed high expression levels in later stages (II and III) of sweet cherry fruit development and played a consistent role in cuticular lipid and anthocyanin biosynthesis.

Other processes and characteristics related to fruit quality include fruit development, the potential for fruit storage and the ripening process. Several TFs have been linked to the control of these events, including AP2/ERF; SBP (CNR); bZIP; NAC; HD-ZIP; ARF (and the ARF regulating proteins AUX/IAA); EIL; and LIM (Trainotti et al., 2007; Ziliotto et al., 2008; Ziosi et al., 2008; Soto et al., 2012; Liang et al., 2013; Lovisetto et al., 2013; Pirona et al., 2013). Shigyo et al. (2006) described that the AP2/ERF TFs act under several biological processes and that these TFs may play an important role in fruit growth and development in climacteric fruits (i.e., peaches, nectarines, and Japanese plums), especially in the ethylene signal transduction pathway. ERFs are plant-specific, nucleus-localized proteins. They serve as TFs that bind conserved motifs in promoter regions of target genes (Zhang et al., 2012), providing a route for ethylene signal activation at the level of target gene transcription, suggesting the involvement of ERFs in the ripening process of climacteric fruits (El-Sharkawy et al., 2009). The peach transcript model ppa010982m, similar to the *ETHYLENE RESPONSIVE ELEMENT BINDING FACTOR 4* (*ERF4*) from *Arabidopsis*, has already been proposed as a candidate gene for fruit maturation date in different climacteric *Prunus* species (Dirlewanger et al., 2012).

Fruit development results in an increase in size through both cell division and expansion. The *SBP* genes were first characterized as *SQUAMOSA* binding proteins (*SBPs*) that regulate the expression of *MADS-BOX* genes in early flower development (Klein et al., 1996), and they also play a critical role in regulating flowering in addition to affecting fruit development (Manning et al., 2006). An exhaustive analysis of *SBP* tomato gene expression revealed that a large proportion of members were ubiquitously and constitutively expressed (from seedling to ripe fruit), while other members showed a more differentiated expression overall (Salinas et al., 2012). In particular, transcripts of *SlySBP12b*, *SlySBP10* and *CNR* were highly accumulated in ripe fruit. In peach, the gene ppa022739m that codes for a putative TF containing the Squamosa-Promoter Binding Protein (SBP) domain is located in the region in which the major QTL controlling fruit maturation time was mapped (Romeu et al., 2014). On the same locus, the NAC1 ppa008301 has been proposed as a candidate for controlling the harvest date (Pirona et al., 2013).

The involvement of the bZIP gene family in fruit ripening in apricot and peach has also been described by Manganaris et al. (2011). These authors identified different contigs showing homology to the protein phosphatase PP2C family members, such as ABI1 and ABI2, which were only differentially expressed in apricot. *ABI1* has been considered as a negative regulator of ABA signaling, and in apricot ripening, the expression of *PP2C* members was higher than in peach, suggesting a lower sensitivity of apricot to ABA. ABA signaling is linked to the gene *Contig298*, which, besides being homologous to the TF *ATB2/bZIP11* belonging to the bZIP family, is also transcribed more abundantly in ripe peach and immature apricot. In the fleshy fruit of apricot and peach, however, the *ATB2/bZIP11* function and the relationship with ABA are not clear. Furthermore, the exogenous application of jasmonates (JAs) during fruit ripening altered the level of transcripts of *bZIP* contig298, which is associated with developmental regulation (Soto et al., 2012). According to Lovisetto et al. (2013), the dilated ripening and the enhanced metabolism of tomato fruit over-expressing the peach *bZIP* gene suggests that this gene might participate in ripening regulation, but its molecular action remains unknown.

Fresh fruit quality is closely linked to the control of senescence of the fruit. The *NAC* TFs were derived from the names of three proteins: NAM (no apical meristem), ATAF1-2, and CUC2 (cup-shaped cotyledon) (Souer et al., 1996). These TFs were initially recognized as factors implicated in various processes of plant development, such as in the response to pathogens and viral infections. More recently, the NAC TFs have been reported to play an essential role in regulating cell division and cell senescence. In the analysis of two populations of peach, segregating for a maturity date locus, Pirona et al. (2013) identified a variant *NAC* gene (*PpNAC1*, ppa008301m) on chromosome 4 that was shown to co-segregate with the fruit maturity locus, suggesting this gene as a candidate for controlling ripening time in peach. This gene has been shown to interact with a second NAC, mapped on chromosome 5 and named BLOOD (BL), because it is responsible for the blood-flesh trait (Zhou et al., 2015). The heterodimer BL/PpNAC1 transactivates the expression of the abovementioned *PpMYB10.1* gene, thus leading to anthocyanin production in the mesocarp of *BL/BL* and *BL/bl* genotypes. The activity of the BL/PpNAC1 heterocomplex is repressed by PpSLP1, an SBP encoded by a gene whose expression ceases at ripening, thus allowing *PpMYB10.1* transcription and the resulting anthocyanin accumulation (Zhou et al., 2015). In sweet cherry, Alkio et al. (2014) also identified an *NAC* gene related to fruit ripening.

The involvement of ARFs and their cognate proteins (Aux/IAA proteins) in peach and apricot fruit ripening has been widely studied (Trainotti et al., 2007; Bonghi et al., 2011; Manganaris et al., 2011). Nonetheless, only four of the 17 ARF genes present in the peach genome have been studied. The role of these TFs in the early phases of fleshy fruit development must therefore be investigated, just as it has recently been deeply examined in tomato (Zouine et al., 2014). In fact, the expression of tomato ARFs has been found to sharply increase upon pollination/fertilization. Given the role of auxin signaling in the fruit set process (De Jong et al., 2009; Devoghalaere et al., 2012), the dynamics of the expression pattern of tomato ARFs is indicative of their putative involvement in mediating auxin responses during the flower-to-fruit transition. Genome-wide expression profiling using RNA-Seq has revealed that tomato *ARF* genes are regulated by both ethylene and auxin, suggesting the potential contribution of these genes to the convergent mechanism between the signaling pathways of these two hormones. To reinforce this theory of co-operation between auxin and ethylene in the control of fruit ripening, it is worthy to note that the ppa003113m gene, similar to *ETHYLENE-INSENSITIVE3-LIKE 3* (*EIL3*), together with genes related to auxin synthesis and *ARFs* (ppa002986m, ppa001557m, and ppa002082m), has been located in a region containing a peach QTL associated with fruit ripening time (chromosome 6) (Romeu et al., 2014). It is worth remembering that the expression of softening-related genes, such as *Endopolygalacturonase* (*PpPG*) and *Expansin3* (*PpExp3*) in peach fruits, is regulated by ethylene at the transcriptional level (Hayama et al., 2006) and is required for the progression of the fruit softening process. Fruit softening is essential for fruit quality, yet the control of this process is very important for extending the shelf life of post-harvest fruits, especially in peach, nectarine, plum, and apricot.

In peach, the *LIM* gene has also been associated with changes in firmer flesh that contribute to the regulation of the cell wall structure under signaling by MJ. In peach fruits where JAs were exogenously applied, Ziosi et al. (2008) identified a ripening delay due to a possible interference with ripening and stress-related genes. It was supposed that LIM TF may alter the phenylpropanoid pathway in MJ-treated fruit, leading to an accumulation of lignin precursors, contributing to cell wall strengthening and acting in the process of delaying ripening in peaches. In the case of *Prunus* species, lignification is a crucial event during fruit development, taking into account that the fruit is a drupe. Endocarp lignification plays a critical role from a practical point of view, because peach varieties showing a phenotype called “split pit,” where the endocarp does not seal along the suture, have seeds that are more vulnerable to pests and disease.

On the other hand, during early phases of peach fruit development, simultaneous activation of the lignin and flavonoid pathways in the mesocarp and endocarp has been detected (Dardick et al., 2010; Hu et al., 2011), while in later phases, high spatial specificity in terms of transcripts, protein and metabolite accumulation has been observed. In fact, in the endocarp, the activation of genes involved in the lignin pathway is accompanied by a repression of genes responsible for flavonoid metabolism, while in the epicarp and mesocarp, these two pathways are regulated in the opposite manner (Dardick and Callahan, 2014). This result suggests that drupe patterning is controlled by a highly coordinated gene network. On the basis of expression profile data, it can be seen that a pivotal role is played in this network by the same TFs that control dehiscence in *Brassica* species (Dardick et al., 2010). Dardick et al. observed that the expression of the peach homologs of *SHATTERPROOF* (*SHP*), *SEEDSTICK* (*STK*), and *FRUITFUL* (*FUL*), three *MADS-BOX* genes, was spatially controlled and was restricted to the endocarp for *SHP* and *STK*, while *FUL* transcript accumulation was higher in the mesocarp but constitutively low in the endocarp. This observation is consistent with the theory of a possible role of these genes in delimiting endocarp lignification margins, as demonstrated in the *Arabidopsis thaliana* silique (Ferraìndiz et al., 2000). Furthermore, Tani et al. (2007) found that *SHP* expression in a split pit resistant variety was lower during the lignification stage, while *FUL* expression was significantly elevated in the sensitive variety during later stages of fruit growth.

In the endocarp, expression of *SHP* and *STK* is higher until the onset of lignin accumulation. Later on, this event is paralleled by the expression of a peach homolog of *NST1* (*No Secondary wall Thickening*), an NAC TF that rapidly accumulates along with secondary metabolism and cell wall biosynthesis genes, as observed in *Arabidopsis* (Mitsuda et al., 2005). In addition to these genes, the expression of a peach homolog of *SPATULA* (*SPT*), a bHLH TF involved in the control of siliqua valve identity (Groszmann et al., 2011), is consistent with a role in specifying endocarp margins (Tani et al., 2011). Collectively, these data imply that highly similar pathways likely control development in both *Prunus* and *Brassica* fruits. Studies of this characteristic raise two contrasting perspectives. On the one hand, the “split pit” phenotype is not wanted because it enhances seed vulnerability, and furthermore, for canning peach cultivars, the “split pit” also creates problems during industrial peach processing. On the other hand, however, while some peach cultivars have a high percentage of germination without mechanically cracking the endocarp, in some peach rootstocks, the endocarp suture is so adhered/lignified that it creates a physical barrier, and seed germination is drastically reduced without mechanical endocarp cracking to release the seeds followed by stratification. To further complicate the situation, the *SHP/PLENA* peach gene is involved not only in flower development but also in the activation of the ripening process by regulating the expression of ripening-related genes (Tadiello et al., 2009). This is similar to the role played by *TAGL1*, the tomato ortholog of *SHP/PLENA* (Vrebalov et al., 2009). Fine-tuning the functions in which this peach gene is involved is therefore a demanding task for breeders.

## Resistance to biotic stresses

Breeding for pest and disease (biotic stress) resistance is another important breeding objective in *Prunus*. In this sense, knowledge about the molecular basis of resistance to different pathogens and the role of the different TFs in this process is of critical interest in the development of efficient breeding strategies and markers for selection.

The involvement of the pathogen resistant genes *PR1* (*Pp-PR1a*, *Pp-PR1b*) and three *PR5*s (*Pp-TLP1*, *Pp-TLP2*, and *Pp-TLP3*) in the resistance to *Xanthomonas arboricola pv. pruni* in peach was investigated by Sherif et al. (2012), who verified an induction of PR genes in response to bacterial infection. In this case, the interaction of both signaling molecules and TF *MYC2* (JA signaling), *ERF* (JA/ET signaling), *WRKY* (SA signaling), and *TGA* (SA signaling), is determinant in mediating resistance against this pathogen. Lee et al. (2010) investigated the role of the *CUTINASE* gene (*MfCUT1*) in wild-type (WT) and *MfCUT1*-overexpressing transformants of *M. fructicola* and described several TFs that may be involved in the redox regulation of *MfCUT1* expression. The presence of NIT2 in the *MfCUT1* promoter region indicates a possible effect of starvation as a form of nitrogen limitation that may regulate *MfCUT1* expression, because NIT2 is a nitrogen metabolic regulator and mediates the repression of its target genes when primary nitrogen sources are available. Another TF involved in the response to *MfCUT1* expression is an AP-l protein (Activator Protein) that has been linked to signal transduction pathways coupled with oxidative stress.

In peach leaves inoculated with *X. arboricola pv. pruni*, Sherif et al. (2013) also identified three genes that encode *ERF* repressors, *PpERF12*, *PpERF3a*, and *PpERF3b*, which showed higher induction in the susceptible peach genotype evaluated than in the resistant one. These results suggest a negative role for these genes in disease resistance. In additional analyses, transgenic *Nicotiana tabacum* plants overexpressing *PpERF3baΔ*EAR showed less disease symptoms than either plants overexpressing the full-length gene or WT plants, suggesting that the resistance of *PpERF3baΔ*EAR plants is associated with the enhanced induction of pathogenesis-related *(PR)* genes.

In addition, the transcriptome analysis of peach leaves inoculated with *X. arboricola pv pruni* revealed a total of six potential ERF TFs, but only one was up-regulated at 2 h post-inoculation (hpi) (Socquet-Juglard et al., 2013). Furthermore, the gene ppa006485m, which is similar to a gene encoding a Mitogen-activated protein kinase kinase kinase (MAPKKK15), was down-regulated, while the genes *ppa015973m* and *ppa018075m*, which could putatively belong to the MYB and WRKY family TFs, respectively, were both up-regulated at 12 hpi. These authors also identified three genes similar to bHLH TFs. One of these genes (*ppa017640m*) was differentially expressed at 2 hpi, and two (*ppb012603m* and *ppa022385m*) were differentially expressed at 12 hpi. Furthermore, another four genes (*ppa012687m*, *ppa012737m*, *ppa012242m*, and *ppa011359m*) belonging to zinc finger families were identified and linked to basal defense against pathogen attacks.

To look at another example, the root-knot nematode (RKN) belonging to the *Meloidogyne* genus is among the parasites that cause the greatest damage to the roots of *Prunus* trees around the world. In “Myrobalan plum” rootstocks (*P. cerasifera*), the *Ma* gene that confers complete-spectrum resistance to RKN was cloned and characterized by Claverie et al. (2011) as a *TNL1* (*TIR-NBS-LRR)* gene, which contains five post-LRR (PL) exons and a conserved core motif [CG(a)RL(a)Y], similar to the WRKY transcription factor motif (WRKYGQK) from *RRS1* identified by Deslandes et al. (2002). The similarity of the PL domains of TNL1 to the WRKY TFs implied that the key targets of the RKN species could be WRKY TFs (Claverie et al., 2011), due to their role as central components of many aspects of the innate immune system of the plant in addition to their basal effects on defense, systemic acquired resistance and plant development (Rushton et al., 2010). The new discoveries about TNL-WRKY protein involvement in plant pathogen resistance open up the possibility of identifying more RKN resistance genes in different *Prunus* species.

Finally, *Plum pox virus* (PPV, sharka disease) has been the most studied virus affecting *Prunus* species. From the genomics point of view, Zuriaga et al. (2013) identified *TRAF* transcriptional regulators as the genes responsible for resistance in apricot. More recently, Rubio et al. (2015) demonstrated that early PPV infection in peach leaves was associated with an induction of TFs related to pathogen resistance by jasmonic acid (JA). The increase in JA levels leads to a degradation of JAZ proteins and then to the depression of *MYC2* (and its redundant homologs *MYC3* and *MYC4*), bHLH TFs that play a central role in JA signaling, resulting in the transcriptional activation of downstream target genes (Katsir et al., 2008). Rodamilans et al. (2014) also described the role of the previously mentioned *NBS-LRR* genes in the hypersensitive response to PPV in Japanese plum.

## Resistance to abiotic stresses

Drought, salinity and low temperatures are considered the most important abiotic stresses limiting fruit production and quality in *Prunus*. Identifying the genes and TFs related to these abiotic stresses, and understanding how gene expression is controlled under these conditions, could represent an important contribution for better managing plants, as well as for reducing the negative impact these stresses cause in fruit production. According to Eldem et al. (2012) the responses to drought stress are regulated at the transcriptional and post-transcriptional levels, and miRNAs have been identified as important gene regulators at post-transcriptional levels. These researchers characterized different miRNAs whose targets were various TF genes [*NFYA* (miR169) and *DRE* (miR169)] involved in plant responses to drought. NF-Y TFs are represented by NF-YA, NF-YB, and NF-YC families (Siefers et al., 2009). In *Zea mays*, Nelson et al. (2007) verified that the overexpression of *NF-YB* genes enhanced drought resistance. Li et al. (2008) described that *NF-YA5* reduces anthocyanin production and stomata aperture, and control of stomata movement is an important mechanism for plants to control loss of water from the leaves during drought stress and to avoid dehydration. Alimohammadi et al. (2013) unraveled the interaction between protein AFC2 kinase and nuclear RNA splicing proteins (including SR45, SR33, SRZ-22, and RSZP21), which are involved in the sugar-mediated signaling pathway as well as in the epigenetic response via histone phosphorylation in the resistance to water deficit in wild almond *P. scoparia*. Interestingly, promoter analysis showed differentially expressed genes harboring binding sites of MYB1 and MYB TFs, which are involved in the dehydration response through the ABA signaling pathway.

Cold stress causes tissue injury, delay in growth and reduction in photosynthesis. Plants respond to low temperatures by altering the expression of thousands of genes including TFs (Chinnusamy et al., 2007). In almond, Barros et al. (2012b) showed that a progressive increase in the transcript abundance of *PdCBF2* (*Prunus dulcis* C-repeat binding factor) during autumn was closely related to cold acclimation. The AP2 domain is also considered a regulatory element that stimulates transcription in response to low temperatures in plants (Díaz-Martín et al., 2005). The *CBF* genes belong to the AP2/EREBD multigene superfamily of TFs, and their relationship to cold response and acclimation in *Prunus* species is well-documented (Tittarelli et al., 2009; Barros et al., 2012a,b; Trainin et al., 2013). *CBF* genes are considered key regulators of cold acclimation, and the overexpression of *CBF 1, 2*, or *3* is capable of improving freezing tolerance in *A. thaliana* plants (Owens et al., 2002). They cloned a *CBF1*-ortholog gene of *Fragaria* × *ananassa* (*FaCBF1*) and *P. cerasus* (*PcCBF1*), and the mRNA levels were up-regulated in the leaves of both crops following exposure to 4°C for a period of between 15 min up to 24 h. In the receptacles of two *CaMV35S-CBF1*-transgenic lines of *Fragaria* × *ananassa* “Honeoye,” no significant changes in freezing tolerance were observed in comparison to wild-type plants. Nevertheless, the temperatures at which 50% electrolyte leakage occurred in detached leaf discs from the two transgenic lines were −8.2°C and −10.3°C, respectively, suggesting the influence of the *FaCBF1* gene in cold acclimation. Kitashiba et al. (2002) isolated three *DREB1/CBF*-like genes from *P. avium* L. (sweet cherry), but only the expression of the *D2* genes was found to be induced at low temperature. In *Prunus mume*, Zhang et al. (2013) also identified two *CBF* genes (*PmCBFa* and *PmCBFb*), homologs of the sweet cherry *PaDREB* gene, which were induced at low temperature. Mousavi et al. (2014) observed that the CBF/DREB1 TF was highly expressed in the ovaries of *P. dulcis* under freezing conditions, while no significant alteration in expression was observed in anthers, reinforcing the regulatory involvement of this TF family in cold acclimation.

Just as WRKY TFs have been linked to biotic stress responses as described above, they have also been associated with abiotic stress responses, like high salt or heat levels, osmotic stress, high CO_2_ levels, high ozone concentrations, and cold or drought. When plants are exposed to these abiotic stress situations, WRKY TFs form part of the signaling processes associated with transcriptional reprogramming, acting as negative or positive regulators (Rushton et al., 2010; Chen et al., 2012). Several WRKY proteins have been shown to be involved in plant drought and salinity stress responses (Golldack et al., 2011). In rice, the overexpression of the *OsWRKY11* gene under the control of the HSP101 promoter has been shown to lead to enhanced drought tolerance and to increase the survival rate of green plant parts (Wu et al., 2009). In *A. thaliana*, the *WRKY25* and *WRKY33* genes have been shown to be responsive to both osmotic and oxidative stress. The down-stream regulated target genes of *WRKY33* include transcripts with a role in ROS detoxification, such as peroxidases and glutathione-S-transferases (Jiang and Deyholos, 2009), suggesting that WRKY factors play a role as key regulators in both osmotic and oxidative stress adaptation (Golldack et al., 2011). Interaction between the WRKY TFs and an ethylene response transcriptional co-activator (ERTCA) has also been identified. This interaction was specifically induced during a combination of drought and heat shock in tobacco (Rizhsky et al., 2002), which suggests that this combination is accompanied by the activation of a unique genetic program that differs from the programs activated in plants during either drought or heat shock alone.

## TFs and miRNAs are coordinated in the regulation of target genes involved in organ development and response to abiotic stresses

Studies on miRNAs have demonstrated that TFs are one of the main targets of these genes (Molesini et al., 2012). Different miRNAs target transcripts encoding TFs controlling plant development and are involved in the abiotic stress response (Xia et al., 2012).

Computational studies indicate that miRNAs and TFs appear to form a complex regulatory network with their target genes. These two regulatory circuits are strongly related, allowing for the coordination between the transcriptional and post-transcriptional control of their target genes (Cui et al., 2007). In fact, genes with more TF-binding sites have a higher probability of being targeted by miRNAs and have more miRNA-binding sites on average. In this context, the identification of miRNA targets via high-throughput degradome library sequencing, in addition to the identification of transcription factor binding sites (TFBSs) in the promoter region of target genes, can contribute to our understanding of developmental processes. In the case of the peach model species, this approach is feasible due to the availability of a high quality genome sequence, which makes extensive study of promoter regions and miRNA targets obtained from experimental procedures feasible. Using a degradome approach, Luo et al. (2013) identified 259 miRNA targets in peach, among which about 35% were TFs. It is worthy to note that MiR156 and MiR157, two conserved miRNAs, not only targeted SBP TF, but also targeted genes encoding protein associated with energy metabolism, glucose metabolism, redox status, and ion transport. The expression of many peach miRNAs is tissue-specific or developmental stage-specific (Gao et al., 2012a; Luo et al., 2013), suggesting coordination with TFs in the regulation of miRNA target expression, as observed in mammalian cells (Tan et al., 2008). Zhu et al. (2012) identified in peach three miRNAs that collectively target 49 MYBs, 19 of which are known to regulate phenylpropanoid metabolism, a key pathway associated with stone hardening and fruit color development, highlighting a critical role for miRNAs in the regulation of fruit development and ripening.

miRNAs and TFs have been claimed to be responsible for the high fluctuation in the expression profile of protein-coding genes in response to drought at the transcriptional and post-transcriptional levels (Sunkar et al., 2012; Nakashima et al., 2014). A genome-wide identification of miRNAs associated with drought in peach has made it possible to identify miRNAs targeting mainly TFs and transporters that are differently expressed in leaves and roots subjected to water stress (Eldem et al., 2012). These results reinforce the fact that the miRNA-TF regulatory network can differ among tissues. A similar approach has been used to identify miRNAs associated with the chilling response (Barakat et al., 2012). Several of the miRNAs identified in this case were induced in winter buds and co-localized with QTLs for chilling requirement and bloom date, thus making their gene targets potential candidates for mediating plant responses to cold stress.

## New breeding opportunities

The post-genomic era in *Prunus* species, as well as in other plant species, is characterized by two elements that can cause a paradigmatic shift in the existing approaches: the development of complete reference genomes and the introduction of new methods of high-throughput sequencing of both DNA (DNA-Seq) and RNA (RNA-Seq) (Martínez-Gómez et al., 2012).

At this moment, only two complete reference genomes have been developed in *Prunus*. The IPGI (International Peach Genome Initiative) has released the complete peach genome sequence [peach genome (v1.0)], consisting of eight pseudomolecules (scaffold_1 to 8) representing the eight peach chromosomes accounting for up to 96% of the peach sequences (227.3 Mb) (Verde et al., 2013). This species presents important agronomical and molecular advantages, including self-compatibility, a short juvenile phase and a small genome size, which make it suitable as a model within the *Prunus* genus and the *Rosaceae* family (Jung et al., 2012; Verde et al., 2013). In addition, Zhang et al. (2012) assembled a 280 Mbp genome of Japanese apricot, anchoring 83.9% of the scaffolds to eight chromosomes.

The availability of these complete reference genomes (mainly the peach reference genome) presents one of the most interesting molecular opportunities for the identification of candidate genes linked to agronomic traits and for promoter identification from genomic data. It is now possible to locate the closest markers or candidate genes (including TFs) identified as associated with different QTLs linked to agronomic traits in the reference genome. These new opportunities are of particular interest in the case of *Prunus*, where knowledge concerning the link between genes and agronomic traits remains limited (Salazar et al., 2014).

With the genome sequences available, some strategies that could be used in the functional analysis of *Prunus* TFs include SNP genotyping assays and Genotyping by Sequencing. High-throughput SNP tools have recently been developed in *Prunus* species. In peach a 9K SNP array was developed using only exonic SNPs (Verde et al., 2012), while both exonic and intronic SNPs were used to construct the 6K cherry SNP array (Peace et al., 2012). In apricot a first approach to developing SNP markers combining RNA sequencing and SNPlex™ high-throughput genotyping technology has been recently described, and a significant decrease in the time and cost of genotyping has been estimated (Salazar et al., 2015). Some of these SNPs have already been located inside TF sequences. Due to their high abundance, SNP markers allow us to cover a large proportion of the genome and are ideal for mapping.

The selected genes and TFs can be blasted against the genomic sequences of peach and *A. thaliana* in the Phytozome database (http://www.phytozome.net/) to determine the corresponding orthologous genes/sequences in these genomes. The 1500 bp upstream of the transcriptional start point of the corresponding genes in peach and *Arabidopsis* genomes can then be extracted and considered as promoters. The upstream regions of the selected genes can be analyzed using PLANTPAN (http://plantpan.mbc.nctu.edu.tw) to predict TFs that can activate the selected genes. PLANTPAN finds the TFBs (regulatory elements) on the promoter regions of genes, and, based on the shared TFBs, predicts which TFs might bind/activate all or a majority of the considered genes. The location of the TFs in the peach reference genomes represents an additional advantage, because the gene functions are known. This fact could greatly facilitate the isolation of genes via QTL map-based cloning in the different *Prunus* species following the association of these TFs with the identified QTLs (Salazar et al., 2014) using peach as model species (Verde et al., 2013).

**Figure 3:**
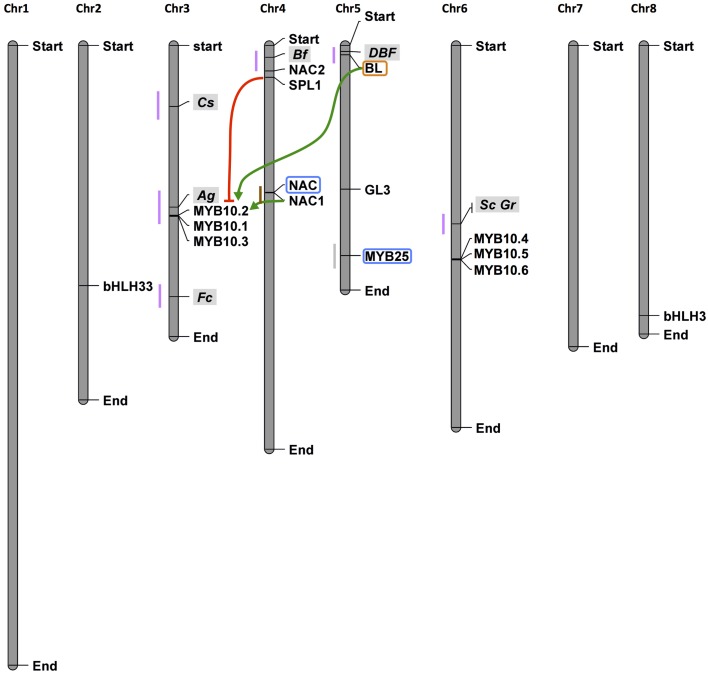
**Localization on the peach genome map of loci and transcription factors controlling fruit traits**. Fruit color is controlled by several loci within some of which TFs have been demonstrated to be the genetic determinant of the trait such as for *BL* in the *DBF* locus on top of chr5. BL interacts with NAC1 to positively (green arrows) regulate MYB10.1 The BL/NAC1 complex is repressed by SPL1 (red line) thus blocking MYB10.1 transcription. MYB10.1, forming a complex with bHLH3 and 33, positively regulate the transcription of structural genes in the flavonoid/anthocyanin pathway, determining pigments accumulation. Closed to *NAC1*, an additional NAC TF has been shown to contribute to the control of maturity date. On chr5 is located *MYB25* that controls the peach/nectarine trait. Genetic markers developed on, or closely to the TF sequences are highlighted by the blue and orange boxes, respectively. These markers have been used to demonstrate that the genes are under the traits, thus will be used for breeding. Similarly, other TFs known be directly involved in the biological process responsible for a trait could be used to develop new genetic markers. Vertical gray bars represent the eight peach chromosomes. Small colored bars represent loci, which names are highlighted in gray, controlling color (purple), maturation date (brown), and peach/nectarine (light gray) traits. Loci for color are: *Cs*, flesh color around the stone; *Ag*, anther color; *Fc*, flower color; *Bf*, blood flesh; *DBF*, dominant blood flesh; *Sc*, fruit skin color; *Gr*, leaf color.

On the other hand, the high level of performance of new methodologies (“high-throughput” or “next generation” NGS) for DNA sequencing (DNA-Seq, in 2005) and the generation of cDNA from RNA (RNA-Seq, in 2008) have also been causing a revolution in biological research. In this context, the functional domains of TF genes can be used for developing informative genic microsatellite markers, such as those obtained in tomato and pepper (Yu et al., 2010) and chickpea (Kujur et al., 2013). These markers, designed transcription factor gene-derived microsatellite (TFGMS) and transcription factor functional domain-associated microsatellite (TFFDMS) markers, can be used in the high-throughput genotyping of new *Prunus* accessions. Moreover, DNA-Seq technology allows for easier resequencing of genotypes (Jackson et al., 2011), assuming a reference-like genome, in the identification of new TFs in different species. TF gene-derived markers are already a reality in peach. They have been used, for example, for the selection of nectarine- or peach-type fruits on the basis of the MYB25 sequence (Vendramin et al., 2014) for which a co-dominant functional diagnostic marker (indelG) has been proposed; for the selection of fruit maturity date using the NAC1 sequence variants (Pirona et al., 2013); and for red flesh color, on the basis of the marker linked to the BL allele (Zhou et al., 2015) (Figure 3). Moreover, other TFs are very good candidates for being the genetic determinants of other traits, such as an SBP and DAMS in QTLs controlling fruit maturation (on LG4, Romeu et al., 2014) and bud dormancy (LG1, Fan et al., 2010), respectively.

Finally, the well-known synteny among *Prunus* and *Rosaceae* genomes (Jung et al., 2009) and transcriptomes (Martínez-Gómez et al., 2011) offers additional molecular opportunities for the analysis of TFs linked to agronomic traits. We can consider the *Prunus* genus as a single gene pool (Jung et al., 2009). In this regard, it is important to note the transferability of molecular information about TFs identified in the different *Prunus* species. This synteny has already been studied in *Prunus* in relation to other genera inside the *Rosaceae* family (Jung et al., 2012). This synteny can result in homologous TFs from a common ancestral DNA including orthologous genes from different species or paralogous genes involving new functions (Shulaev et al., 2008).

## Author contributions

VB, IV, and PM participated in the coordination of the study. MR collected and revised the information about resistance biotic stress. IV collected and revised the information about flowering and bud dormancy. VB and PM collected and revised the information about abiotic stress resistance. LT and CB collected and revised the information about fruit and seed development and miRNAs.

### Conflict of interest statement

The authors declare that the research was conducted in the absence of any commercial or financial relationships that could be construed as a potential conflict of interest.

## Supplementary material

Table S1 **Plant transcription factor (TF) families of peach, Arabidopsis, and poplar described to date in the PlantTFDB database (http://planttfdb.cbi.pku.edu.cn/)**.

Table S2 **Distribution of identified transcription factors in the peach v1.0 reference genome (http://www.rosaceae.org/species/prunus/prunus_persica)**. In each scaffold bold letters indicate alternative transcripts of the same protein coding gene. In addition, the correct v2.0 (http://www.rosaceae.org/gb/gbrowse/prunus_persica_v2.1) pseudomolecules where these genes are located, according to the assembly refinements described in Verde et al. (2013, 2015), are indicated in brackets.

## References

[B1] Alimohammadi A. Shiran B. Martínez-Gómez P. Ebrahimie E. (2013). Identification of water-deficit resistance genes in wild almond *Prunus scoparia* using cDNA-AFLP. Sci. Hort. 159, 19–28. 10.1016/j.scienta.2013.04.023

[B2] Alkio M. Jonas U. Declercq M. Van Nocker S. Knoche M. (2014). Transcriptional dynamics of the developing sweet cherry (*Prunus avium* L.) fruit: sequencing, annotation and expression profiling of exocarp-associated genes. Hort. Res. 1:11 10.1038/hortres.2014.11 PMC4591669 26504533

[B3] An L. Lei H. Shen X. Li T. (2012). Identification and characterization of PpLFL, a homolog of *FLORICAULA/LEAFY* in peach. Plant Mol. Biol. Rep. 30, 1488–1495. 10.1007/s11105-012-0459-x

[B4] Andreotti C. Ravaglia D. Ragaini A. Costa G. (2008). Phenolic compounds in peach (*Prunus persica*) cultivars at harvest and during fruit maturation. Ann. Appl. Biol. 153, 11–23. 10.1111/j.1744-7348.2008.00234.x

[B5] Ariel F. D. Manavella P. A. Dezar C. A. Chan R. L. (2007). The true story of the HD-Zip family. Trends Plant Sci. 12, 419–426. 10.1016/j.tplants.2007.08.003 17698401

[B6] Artlip T. S. Wisniewski M. E. Bassett C. L. Norelli J. L. (2013). CBF gene expression in peach leaf and bark tissues is gated by a circadian clock. Tree Physiol. 33, 866–877. 10.1093/treephys/tpt056 23956128

[B7] Arús P. Verde I. Sosinski B. Zhebentyayeva T. Abbott A. G. (2012). The peach genome. Tree Genet. Genomes 8, 531–547. 10.1007/s11295-012-0493-8

[B8] Atkins J. F. Gesteland R. F. Cech T. R. (2011). RNA Worlds: From Life's to Diversity in Gene Regulation. New York, NY: Cold Spring Harbor; Cold Spring Harbor Laboratory Press.

[B9] Barakat A. Sriram A. Park J. Zhebentyayeva T. Main D. Abbott A. (2012). Genome wide identification of chilling responsive microRNAs in *Prunus persica*. BMC Genomics 13:481. 10.1186/1471-2164-13-481 22978558 PMC3463484

[B10] Barros P. M. Goncalves N. Saibo N. J. M. Oliveira M. M. (2012b). Cold acclimation and floral development in almond bud break: insights into the regulatory pathways. J. Exp. Bot. 63, 4585–4596. 10.1093/jxb/ers144 22685307

[B11] Barros P. M. Gonçalves N. Saibo N. J. M. Oliveira M. M. (2012a). Functional characterization of two almond C-repeat-binding factors involved in cold response. Tree Physiol. 32, 1113–1128. 10.1093/treephys/tps067 22832014

[B12] Bassett C. L. Wisniewski M. E. Artlip T. S. Richart G. Norelli J. L. Renaut J.. (2009). Comparative expression and transcript initiation of three peach dehydrin genes. Planta 230, 107–118. 10.1007/s00425-009-0927-1 19360436

[B13] Ben Mahmoud K. Delporte F. Muhovski Y. Elloumi N. Jemmali A. Druart P. (2013). Expression of *PiABP19, Picdc2* and *PiSERK3* during induction of somatic embryogenesis in leaflets of *Prunus incisa* (Thunb.). Plant Mol. Biol. Rep. 40, 1569–1577. 10.1007/s11033-012-2205-8 23086274

[B14] Bielenberg D. G. Wang Y. Li Z. G. Zhebentyayeva T. Fan S. H. Reighard G. L. (2008). Sequencing and annotation of the evergrowing locus in peach [*Prunus persica* (L.) Batsch] reveals a cluster of six MADS-BOX transcription factors as candidate genes for regulation of terminal bud formation *Tree Genet*. Genomes 4, 495–507. 10.1007/s11295-007-0126-9

[B15] Bonghi C. Trainotti L. Botton A. Tadiello A. Rasori A. Ziliotto F.. (2011). A microarray approach to identify genes involved in seed-pericarp cross-talk and development in peach. BMC Plant Biol. 11:107. 10.1186/1471-2229-11-107 21679395 PMC3141638

[B16] Bouché N. Scharlat A. Snedden W. Bouchez D. Fromm H. (2002). A novel family of calmodulin-binding transcription activators in multicellular organisms. J. Biol. Chem. 277, 21851–21861. 10.1074/jbc.M200268200 11925432

[B17] Byzova M. V. Franken J. Aarts M. G. de Almeida-Engler J. Engler G. Mariani C.. (1999). Arabidopsis STERILE APETALA, a multifunctional gene regulating inflorescence, flower, and ovule development. Genes Dev. 13, 1002–1014. 10.1101/gad.13.8.1002 10215627 PMC316639

[B18] Chen L. Song Y. Li S. Zhang L. Zou C. Yu D. (2012). The role of WRKY transcription factors in plant abiotic stresses. Biochim. Biophys. Acta 1819, 120–128. 10.1016/j.bbagrm.2011.09.002 21964328

[B19] Chinnusamy V. Zhu J. Zhu J. K. (2007). Cold stress regulation of gene expression in plants. Trends Plant Sci. 12, 444–451. 10.1016/j.tplants.2007.07.002 17855156

[B20] Claverie M. Dirlewanger E. Bosselut N. Van Ghelder C. Voisin R. Kleinhentz M.. (2011). The Ma gene for complete-spectrum resistance to meloidogyne species in *Prunus* is a TNL with a huge repeated C-terminal post-LRR region. Plant Physiol. 156, 779–792. 10.1104/pp.111.176230 21482634 PMC3177275

[B21] Cubas P. Lauter N. Doebley J. Coen E. (1999). The TCP domain: a motif found in proteins regulating plant growth and development. Plant J. 18, 215–222. 10.1046/j.1365-313X.1999.00444.x 10363373

[B22] Cui Q. Yu Z. Pan Y. Purisima E. O. Wang E. (2007). MicroRNAs preferentially target the genes with high transcriptional regulation complexity. Biochem. Biophys. Res. Commun. 352, 733–738. 10.1016/j.bbrc.2006.11.080 17141185

[B23] Curaba J. Herzog M. Vachon G. (2003). GeBP, the first member of a new gene family in Arabidopsis, encodes a nuclear protein with DNA-binding activity and is regulated by KNAT1. Plant J. 33, 305–317. 10.1046/j.1365-313X.2003.01622.x 12535344

[B24] Cvitanich C. Pallisgaard N. Nielsen K. A. Hansen A. C. Larsen K. Pihakaski-Maunsbach K.. (2000). CPP1, a DNA-binding protein involved in the expression of a soybean leghemoglobin c3 gene. Proc. Natl. Acad. Sci. U.S.A. 97, 8163–8168. 10.1073/pnas.090468497 10859345 PMC16687

[B25] D'Agostino I. B. Kieber J. J. (1999). Phosphorelay signal transduction: the emerging family of plant response regulators. Trends Biochem. Sci. 24, 452–456. 10542414 10.1016/s0968-0004(99)01465-6

[B26] D'Archivio M. Filesi C. Di Benedetto R. Gargiulo R. Giovannini C. Masella R. (2007). Polyphenols, dietary sources and bioavailability. Ann. Ist. Super. Sanita 43, 348–361. 18209268

[B27] Dardick C. D. Callahan A. M. (2014). Evolution of the fruit endocarp: molecular mechanism underlying adaptions in seed protection and dispersal strategies. Front. Plant Sci. 5:284. 10.3389/fpls.2014.00284 25009543 PMC4070412

[B28] Dardick C. D. Callahan A. M. Chiozzotto R. Schaffer R. J. Piagnani M. C. Scorza R. (2010). Stone formation in peach fruit exhibits spatial coordination of the lignin and flavonoid pathways and similarity to Arabidopsis dehiscence. BMC Biol. 8:13. 10.1186/1741-7007-8-13 20144217 PMC2830173

[B29] De Jong M. Mariani C. Vriezen W. H. (2009). The role of auxin and gibberellin in tomato fruit set. J. Exp. Bot. 60, 1523–1532. 10.1093/jxb/erp094 19321650

[B30] Deslandes L. Olivier J. Theulieres F. Hirsch J. Feng D. X. Bittner-Eddy P.. (2002). Resistance to *Ralstonia solanacearum* in *Arabidopsis thaliana* is conferred by the recessive *RRS1-R* gene, a member of a novel family of resistance genes. Proc. Natl. Acad. Sci. U.S.A. 99, 2404–2409. 10.1073/pnas.032485099 11842188 PMC122377

[B31] Desveaux D. Marechal A. Brisson N. (2005). Whirly transcription factors: defence gene regulation and beyond. Trends Plant Sci. 10, 95–102. 10.1016/j.tplants.2004.12.008 15708347

[B32] Devoghalaere F. Doucen T. Guitton B. Keeling J. Payne W. Ling T. J.. (2012). A genomics approach to understanding the role of auxin in apple (*Malus × domestica*) fruit size control. BMC Plant Biol. 12:7. 10.1186/1471-2229-12-7 22243694 PMC3398290

[B33] Díaz-Martín J. Almoguera C. Prieto-Dapena P. Espinosa J. M. Jordano J. (2005). Functional interaction between two transcription factors involved in the developmental regulation of a small heat stress protein gene promoter. Plant Physiol. 139, 1483–1494. 10.1104/pp.105.069963 16244139 PMC1283783

[B34] Dietrich R. A. Richberg M. H. Schmidt R. Dean C. Dangl J. L. (1997). A novel zinc finger protein is encoded by the Arabidopsis LSD1 gene and functions as a negative regulator of plant cell death. Cell 88, 685–694. 10.1016/S0092-8674(00)81911-X 9054508

[B35] Dirlewanger E. Quero-García J. Le Dantec L. Lambert P. Ruiz D. Dondini L.. (2012). Comparison of the genetic determinism of two key phenological traits, flowering and maturity dates, in three *Prunus* species: peach, apricot and sweet cherry. Heredity 109, 280–292. 10.1038/hdy.2012.38 22828898 PMC3477885

[B36] Du D. L. Hao R. J. Cheng T. R. Pan H. T. Yang W. R. Wang J. (2013). Genome-wide analysis of the AP2/ERF gene family in *Prunus mume*. Plant Mol. Biol. Rep. 31, 741–750. 10.1007/s11105-012-0531-6

[B37] Dubé A. Bisaillon M. Perreault J.-P. (2009). Identification of proteins from *Prunus persica* that interact with peach latent mosaic viroid. J. Virol. 83, 12057–12067. 10.1128/JVI.01151-09 19759139 PMC2786745

[B38] Eldem V. Akcay U. C. Ozhuner E. Bakir Y. Uranbey S. Unver T. (2012). Genome-wide identification of miRNAs responsive to drought in peach (*Prunus persica*) by high-throughput deep sequencing. PLoS ONE 7:e50298. 10.1371/journal.pone.0050298 23227166 PMC3515591

[B39] El-Sharkawy I. Kim W. S. El-Kereamy A. Jayasankar S. Svircev A. M. Brown D. C. W. (2007). Isolation and characterization of four ethylene signal transduction elements in plums (*Prunus salicina* L.). J. Exp. Bot. 58, 3631–3643. 10.1093/jxb/erm213 18057041

[B40] El-Sharkawy I. Sherif S. Mila I. Bouzayen M. Jayasankar S. (2009). Molecular characterization of seven genes encoding ethylene-responsive transcriptional factors during plum fruit development and ripening. J. Exp. Bot. 60, 907–922. 10.1093/jxb/ern354 19213809 PMC2652048

[B41] Eshed Y. Baum S. Perea J. V. Bowman J. L. (2001). Establishment of polarity in lateral organs of plants. Curr. Biol. 11, 1251–1260. 10.1016/S0960-9822(01)00392-X 11525739

[B42] Espley R. V. Hellens R. P. Putterill J. Stevenson D. E. Kutty-Amma S. Allan A. C. (2007). Red colouration in apple fruit is due to the activity of the MYB transcription factor, MdMYB10. Plant J. 49, 414–427. 10.1111/j.1365-313X.2006.02964.x 17181777 PMC1865000

[B43] Eulgem T. Rushton P. J. Robatzek S. Somssich I. E. (2000). The WRKY superfamily of plant transcription factors. Trends Plant Sci. 5, 199–206. 10.1016/S1360-1385(00)01600-9 10785665

[B44] Fan S. Bielenberg D. G. Zhebentyanyeva T. N. Reighard G. L. Okie W. R. Holland D.. (2010). Mapping quantitative trait loci associated with chilling requirement, heat requirement and bloom date in peach (*Prunus persica*). New Phytol. 185, 917–930. 10.1111/j.1469-8137.2009.03119.x 20028471

[B45] Feng J. R. Chen X. S. Yuan Z. H. He T. M. Zhang L. J. Wu Y.. (2006). Proteome comparison following self- and across-pollination in self-incompatible apricot (*Prunus armeniaca* L.). Protein J. 25, 328–335. 10.1007/s10930-006-9018-3 16947077

[B46] Ferraìndiz C. Liljegren S. J. Yanofsky M. F. (2000). Negative regulation of the SHATTERPROOF genes by FRUITFULL during Arabidopsis fruit development. Science 289, 436–438. 10.1126/science.289.5478.436 10903201

[B47] Fridborg I. Kuusk S. Moritz T. Sundberg E. (1999). The Arabidopsis dwarf mutant shi exhibits reduced gibberellin responses conferred by overexpression of a new putative zinc finger protein. Plant Cell 6, 1019–1032. 10.1105/tpc.11.6.1019 10368174 PMC144241

[B48] Fujita A. Kikuchi Y. Kuhara S. Misumi Y. Matsumoto S. Kobayashi H. (1989). Domains of the SFL1 protein of yeasts are homologous to Myc oncoproteins or yeast heat-shock transcription factor. Gene 85, 321–328. 10.1016/0378-1119(89)90424-1 2697640

[B49] Gao Z. H. Luo X. Y. Shi T. Cai B. Zhang Z. Cheng Z. M.. (2012a). Identification and validation of potential conserved microRNAs and their targets in peach (*Prunus persica*). Mol. Cells 34, 239–249. 10.1007/s10059-012-0004-7 22878892 PMC3887836

[B50] Gao Z. H. Shi T. Luo X. Y. Zhang Z. Zhuang W. B. Wang L. J. (2012b). High-throughput sequencing of small RNAs and analysis of differentially expressed microRNAs associated with pistil development in Japanese apricot. BMC Genomics 13:371 10.1186/1471-2164-13-371 22863067 PMC3464595

[B51] Giannino D. Condello E. Bruno L. Testone G. Tartarini A. Cozza R.. (2004). The gene geranylgeranyl reductase of peach (*Prunus persica* [L.] Batsch) is regulated during leaf development and responds differentially to distinct stress factors. J. Exp. Bot. 55, 2063–2073. 10.1093/jxb/erh217 15286145

[B52] Golldack D. Lüking I. Yang O. (2011). Plant tolerance to drought and salinity: stress regulating transcription factors and their functional significance in the cellular transcriptional network. Plant Cell Rep. 30, 1383–1391. 10.1007/s00299-011-1068-0 21476089

[B53] Golz J. F. Hudson A. (1999). Plant development: YABBYs claw to the fore. Curr. Biol. 9, 861–863. 10.1016/S0960-9822(00)80047-0 10574752

[B54] Groszmann M. Paicu T. Alvarez J. P. Swain S. M. Smyth D. R. (2011). SPATULA and ALCATRAZ, are partially redundant, functionally diverging bHLH genes required for Arabidopsis gynoecium and fruit development. Plant J. 68, 816–829. 10.1111/j.1365-313X.2011.04732.x 21801252

[B56] Guo C. Zhang J. Q. Peng T. Bao M. Z. Zhang J. W. (2014). Structural and expression analyses of three PmCBFs from *Prunus mume*. Biol. Plant. 58, 247–255. 10.1007/s10535-014-0393-x

[B57] Halbach T. Scheer N. Werr W. (2000). Transcriptional activation by the PHD finger is inhibited through an adjacent leucine zipper that binds 14-3-3 proteins. Nucleic Acids Res. 28, 3542–3550. 10.1093/nar/28.18.3542 10982874 PMC110726

[B58] Hartmann U. Sagasser M. Mehrtens F. Stracke R. Weisshaar B. (2005). Differential combinatorial interactions of cisacting elements recognized by R2R3-MYB, BZIP, and BHLH factors control light-responsive and tissue-specific activation of phenylpropanoid biosynthesis genes. Plant Mol. Biol. 57, 155–171. 10.1007/s11103-004-6910-0 15821875

[B59] Hayama H. Shimada T. Fujii H. Ito A. Kashimura Y. (2006). Ethylene-regulation of fruit softening and softening-related genes in peach. J. Exp. Bot. 57, 4071–4077. 10.1093/jxb/erl178 17077183

[B60] Hu H. Liu Y. Shi G. L. Liu Y. P. Wu R. J. Yang A. Z.. (2011). Proteomic analysis of peach endocarp and mesocarp during early fruit development. Physiol. Plant. 142, 390–406. 10.1111/j.1399-3054.2011.01479.x 21496031

[B61] Hudson M. Ringli C. Boylan M. T. Quail P. H. (1999). The FAR1 locus encodes a novel nuclear protein specific to phytochrome A signalling. Genes Dev. 13, 2017–2027. 10.1101/gad.13.15.2017 10444599 PMC316922

[B62] Husbands A. Bell E. M. Shuai B. Smith H. M. S. Springer P. S. (2007). LATERAL ORGAN BOUNDARIES defines a new family of DNA-binding transcription factors and can interact with specific bHLH proteins. Nucleic Acids Res. 35, 6663–6671. 10.1093/nar/gkm775 17913740 PMC2095788

[B63] Infante R. Martínez-Gómez P. Predieri S. (2011). Breeding for fruit quality in *Prunus*, in Breeding for Fruit Quality, eds Jenks M. A. Bebeli P. J. (New York, NY: Wiley & Blackwel), 201–229.

[B64] Jackson S. A. Iwata A. Lee S. H. Schmutz J. Shoemaker R. (2011). Sequencing crop genomes: approaches and applications. New Phytol. 191, 915–925. 10.1111/j.1469-8137.2011.03804.x 21707621

[B65] Jiang Y. Deyholos M. K. (2009). Functional characterization of Arabidopsis NaCl-inducible WRKY25 and WRKY33 transcription factors in abiotic stresses. Plant Mol. Biol. 69, 91–105. 10.1007/s11103-008-9408-3 18839316

[B66] Jiménez S. Lawton-Rauh A. L. Reighard G. L. Abbott A. G. Bielenberg D. G. (2009). Phylogenetic analysis and molecular evolution of the dormancy associated MADS-BOX genes from peach. BMC Plant Biol. 9:81. 10.1186/1471-2229-9-81 19558704 PMC2713236

[B67] Jiménez S. Reighard G. L. Bielenberg D. G. (2010). Gene expression of DAM5 and DAM6 is suppressed by chilling temperatures and inversely correlated with bud break rate. Plant Mol. Biol. 73, 157–167. 10.1007/s11103-010-9608-5 20143130

[B68] Jin J. P. Zhang H. Kong L. Gao G. Luo J. C. (2014). PlantTFDB 3.0: a portal for the functional and evolutionary study of plant transcription factors. Nucleic Acids Res. 42, D1182–D1187. 10.1093/nar/gkt1016 24174544 PMC3965000

[B69] Jung S. Cestaro A. Troggio M. Main D. Zheng P. Cho I.. (2012). Whole genome comparisons of *Fragaria*, *Prunus* and *Malus* reveal different modes of evolution between Rosaceous subfamilies. BMC Genomics 13:129. 10.1186/1471-2164-13-129 22475018 PMC3368713

[B70] Jung S. Jiwan D. Cho I. Abbott A. Tomkins J. Main D. (2009). Synteny of *Prunus* and other model plant species. BMC Genomics 10:76. 10.1186/1471-2164-10-76 19208249 PMC2647949

[B71] Karp G. (2008). Cell and Molecular Biology. Danvers, MA: John Wiley & Sons, Inc.

[B72] Katsir L. Schilmiller A. L. Staswick P. E. He S. Y. Howe G. A. (2008). COI1 is a critical component of a receptor for jasmonate and the bacterial virulence factor coronatine. Proc. Natl. Acad. Sci. U.S.A. 105, 7100–7105. 10.1073/pnas.0802332105 18458331 PMC2383947

[B73] Kim J. H. Choi D. Kende H. (2003). The AtGRF family of putative transcription factors is involved in leaf and cotyledon growth in Arabidopsis. Plant J. 36, 94–104. 10.1046/j.1365-313X.2003.01862.x 12974814

[B74] Kirik V. Baumlein H. (1996). A novel leaf-specific myb-related protein with a single binding repeat. Gene 183, 109–113. 10.1016/S0378-1119(96)00521-5 8996094

[B75] Kitashiba H. Matsuda N. Ishizaka T. Nakano H. Suzuki T. (2002). Isolation of genes similar to DREB1/CBF from sweet cherry (*Prunus avium* L.). J. Jpn. Soc. Hort. Sci. 71, 651–657. 10.2503/jjshs.71.651

[B76] Klein J. Saedler H. Huijser P. (1996). A new family of DNA binding proteins includes putative transcriptional regulators of the *Antirrhinum majus* floral meristem identity gene SQUAMOSA. Mol. Gen. Genet. 250, 7–16. 8569690 10.1007/BF02191820

[B77] Kornberg R. D. (2007). The molecular basis of eukaryotic transcription. Proc. Natl. Acad. Sci. U.S.A. 104, 12955–12961. 10.1073/pnas.0704138104 17670940 PMC1941834

[B78] Krishnamurthy S. Hampsey M. (2008). Eukaryotic transcription initiation. Curr. Biol. 19, R153–R156. 10.1016/j.cub.2008.11.052 19243687

[B79] Kujur A. Bajaj D. Saxena M. S. Tripathi S. Upadhyaya H. D. Gowda C. L. L.. (2013). Functionally relevant microsatellite markers from chickpea transcription factor genes for efficient genotyping applications and trait association mapping. DNA Res. 20, 355–374. 10.1093/dnares/dst015 23633531 PMC3738162

[B80] Kumagai T. Ito S. Nakamichi N. Niwa Y. Murakami M. Yamashino T. (2008). The common function of a novel subfamily of B-Box zinc finger proteins with reference to circadian-associated events in *Arabidopsis thaliana*. Biosci. Biotechnol. Biochem. 72, 1539–1549. 10.1271/bbb.80041 18540109

[B81] Lagercrantz U. Axelsson T. (2000). Rapid evolution of the family of CONSTANS LIKE genes in plants. Mol. Biol. Evol. 17, 1499–1507. 10.1093/oxfordjournals.molbev.a026249 11018156

[B82] Lalli D. A. Decroocq V. Blenda A. V. Schurdi-Levraud V. Garay L. Le Gall O.. (2005). Identification and mapping of resistance gene analogs (RGAs) in *Prunus*: a resistance map for *Prunus*. Theor. Appl. Genet. 111, 1504–1513. 10.1007/s00122-005-0079-z 16195885

[B83] Landschulz W. H. Johnson P. F. McKnight S. L. (1988). The leucine zipper: a hypothetical structure common to a new class of DNA binding proteins. Science 240, 1759–1764. 10.1126/science.3289117 3289117

[B84] Lee H. Yoo S. J. Lee J. H. Kim W. Yoo S. K. Fitzgerald H.. (2010). Genetic framework for flowering-time regulation by ambient temperature-responsive miRNAs in Arabidopsis. Nucleic Acids Res. 38, 3081–3093. 10.1093/nar/gkp1240 20110261 PMC2875011

[B85] Leida C. Conesa A. Llacer G. Badenes M. L. Rios G. (2012). Histone modifications and expression of DAM6 gene in peach are modulated during bud dormancy release in a cultivar-dependent manner. New Phytol. 193, 67–80. 10.1111/j.1469-8137.2011.03863.x 21899556

[B86] Leida C. Terol J. Marti G. Agusti M. Llacer G. Badenes M. L.. (2010). Identification of genes associated with bud dormancy release in *Prunus persica* by suppression subtractive hybridization. Tree Physiol. 30, 655–666. 10.1093/treephys/tpq008 20231169

[B87] Li W.-X. Oono Y. Zhu J. He X.-J. Wu J.-M. Iida K.. (2008). The Arabidopsis NFYA5 transcription factor is regulated transcriptionally and posttranscriptionally to promote drought resistance. Plant Cell 20, 2238–2251. 10.1105/tpc.108.059444 18682547 PMC2553615

[B88] Li Z. Thomas T. L. (1998). PEI1, an embryo-specific zinc finger protein gene required for heart-stage embryo formation in Arabidopsis. Plant Cell 10, 383–398. 10.2307/3870596 9501112 PMC143998

[B89] Li Z. Reighard G. L. Abbott A. G. Bielenberg D. G. (2009). Dormancy associated MADS-BOX genes from the EVG locus of peach [*Prunuspersica* (L.) Batsch] have distinct seasonal and photoperiodic expression patterns. J. Exp. Bot. 60, 3521–3530. 10.1093/jxb/erp195 19553369 PMC2724702

[B90] Liang L. Zhang B. Yin X. R. Xu C. J. Sun C. D. Chen K. S. (2013). Differential expression of the CBF gene family during postharvest cold storage and subsequent shelf-life of peach fruit. Plant Mol. Biol. Rep. 31, 1358–1367. 10.1007/s11105-013-0600-5

[B91] Lisso J. Altmann T. Mussig C. (2006). The AtNFXL1 gene encodes a NF-X1 type zinc finger protein required for growth under salt stress. FEBS Lett. 580, 4851–4856. 10.1016/j.febslet.2006.07.079 16905136

[B92] Littlewood T. D. Evan G. I. (1995). Transcription factors 2: helix-loop-helix. Protein Profile 2, 621–702. 7553065

[B93] Liu X. M. Anderson J. M. Pijut P. M. (2010). Cloning and characterization of *Prunus serotina* AGAMOUS, a putative flower homeotic gene. Plant Mol. Biol. Rep. 28, 193–203. 10.1007/s11105-009-0140-1

[B94] Lovisetto A. Guzzo F. Tadiello A. Confortin E. Pavanello A. Botton A.. (2013). Characterization of a bZIP gene highly expressed during ripening of the peach fruit. Plant Physiol. Biochem. 70, 462–470. 10.1016/j.plaphy.2013.06.014 23845825

[B95] Luo X. Gao Z. Shi T. Cheng Z. Zhang Z. Ni Z. (2013). Identification of miRNAs and their target genes in peach (*Prunus persica* L.) using high-throughput sequencing and degradome analysis. PLoS ONE 8:e79090. 10.1371/journal.pone.0079090 24236092 PMC3827290

[B96] Manganaris G. A. Rasori A. Bassi D. Geuna F. Ramina A. Tonutti P. (2011). Comparative transcript profiling of apricot (*Prunus armeniaca* L.) fruit development and on-tree ripening. Tree Genet. Genomes 7, 609–616. 10.1007/s11295-010-0360-4

[B97] Manning K. Tör M. Poole M. Hong Y. Thompson A. J. King G. J.. (2006). A naturally occurring epigenetic mutation in a gene encoding an SBP-box transcription factor inhibits tomato fruit ripening. Nat. Genet. 38, 948–952. 10.1038/ng1841 16832354

[B98] Martin T. Hu M. Labbe H. McHugh S. Svircev A. Miki B. (2006). PpAG1, a homolog of AGAMOUS, expressed in developing peach flowers and fruit. Can. J. Bot. 84, 767–776. 10.1139/b06-031

[B99] Martínez-Gómez P. Crisosto C. Bonghi C. Rubio M. (2011). New approaches to *Prunus* transcriptome analysis. Genetica 139, 755–769. 10.1007/s10709-011-9580-2 21584650

[B100] Martínez-Gómez P. Sánchez-Pérez R. Rubio M. (2012). Clarifying omics concepts, challenges and opportunities for *Prunus* breeding in the post-genomic era. OMICS 16, 268–283. 10.1089/omi.2011.0133 22394278

[B101] Mita S. Nagai Y. Asai T. (2006). Isolation of cDNA clones corresponding to genes differentially expressed in pericarp of mume (*Prunus mume*) in response to ripening, ethylene and wounding signals. Physiol. Plant. 128, 531–545. 10.1111/j.1399-3054.2006.00749.x

[B102] Mitsuda N. Hisabori T. Takeyasu K. Sato M. H. (2004). VOZ: isolation and characterization of novel vascular plant transcription factors with a one-zinc finger from *Arabidopsis thaliana*. Plant Cell Physiol. 45, 845–854. 10.1093/pcp/pch101 15295067

[B103] Mitsuda N. Seki M. Shinozaki K. Ohme-Takagi M. (2005). The NAC transcription factors NST1 and NST2 of Arabidopsis regulate secondary wall thickenings and are required for anther dehiscence. Plant Cell 17, 2993–3006. 10.1105/tpc.105.036004 16214898 PMC1276025

[B104] Molesini B. Pii Y. Pandolfini T. (2012). Fruit improvement using intragenesis and artificial microRNA. Trends Biotechnol. 30, 80–88. 10.1016/j.tibtech.2011.07.005 21871680

[B105] Mousavi S. Alisoltani A. Shiran B. Fallahi H. Ebrahimie E. Imani A.. (2014). *De novo* transcriptome assembly and comparative analysis of differentially expressed genes in *Prunus dulcis* Mill. In response to freezing stress. PLoS ONE 9:e104541. 10.1371/journal.pone.0104541 25122458 PMC4133227

[B106] Nakano T. Suzuki K. Fujimura T. Shinshi H. (2006). Genome-wide analysis of the ERF gene family in Arabidopsis and rice. Plant Physiol. 140, 411–432. 10.1104/pp.105.073783 16407444 PMC1361313

[B107] Nakashima K. Yamaguchi-Shinozaki K. Shinozaki K. (2014). The transcriptional regulatory network in the drought response and its crosstalk in abiotic stress responses including drought, cold, and heat. Front. Plant Sci. 5:170. 10.3389/fpls.2014.00170 24904597 PMC4032904

[B107a] Nam J. de Pamphilis C. W. Ma H. Nei M. (2003). Antiquity and evolution of the MADS-box gene family controlling flower development in plants. Mol. Biol. Evol. 20, 1435–1447. 10.1093/molbev/msg152 12777513

[B108] Nelson D. E. Repetti P. P. Adams T. R. Creelman R. A. Wu J. Warner D. C.. (2007). Plant nuclear factor Y (NF-Y) B subunits confer drought tolerance and lead to improved corn yields on water-limited acres. Proc. Natl. Acad. Sci. U.S.A. 104, 16450–16455. 10.1073/pnas.0707193104 17923671 PMC2034233

[B109] Nole-Wilson S. Krizek B. A. (2000). DNA binding properties of the Arabidopsis floral development protein AINTEGUMENTA. Nucleic Acids Res. 28, 4076–4082. 10.1093/nar/28.21.4076 11058102 PMC113152

[B111] Ohme-Takagi M. Shinshi H. (1995). Ethylene-inducible DNA binding proteins that interact with an ethylene-responsive element. Plant Cell 7, 173–182. 10.1105/tpc.7.2.173 7756828 PMC160773

[B112] Owens C. L. Thomashow M. F. Hancock J. F. Iezzoni A. F. (2002). CBF1 orthologs in sour cherry and strawberry and the heterologous expression of CBF1 in strawberry. J. Amer. Soc. Hort. Sci. 127, 489–494.

[B113] Parcy F. Nilsson O. Busch M. A. Lee I. Weigel D. (1998). A genetic framework for floral patterning. Nature 395, 561–566. 10.1038/26903 9783581

[B114] Parenicová L. Folter S. Kieffer M. Horner D. S. Favalli C. Busscher J.. (2003). Molecular and phylogenetic analyses of the complete MADS-BOX transcription factor family in Arabidopsis: new openings to the MADS-BOX world. Plant Cell 15, 1538–1551. 10.1105/tpc.011544 12837945 PMC165399

[B115] Peace C. Bassil N. Main D. Ficklin S. Rosyara U. R. Stegmeir T.. (2012). Development and evaluation of a genome-wide 6K SNP array for diploid sweet cherry and tetraploid sour cherry. PLoS ONE 7:e48305. 10.1371/journal.pone.0048305 23284615 PMC3527432

[B116] Pérez-Rodríguez P. Riano-Pachon D. M. Guedes Correa L. G. Rensing S. A. Kersten B. Mueller-Roeber B. (2009). PlnTFDB: updated content and new features of the plant transcription factor database. Nucleic Acids Res. 38, D822–D827. 10.1093/nar/gkp805 19858103 PMC2808933

[B117] Petroni K. Tonelli C. (2011). Recent advances on the regulation of anthocyanin synthesis in reproductive organs. Plant Sci. 181, 219–229. 10.1016/j.plantsci.2011.05.009 21763532

[B118] Pirona R. Eduardo I. Pacheco I. Linge C. S. Miculan M. Verde I.. (2013). Fine mapping and identification of a candidate gene for a major locus controlling maturity date in peach. BMC Plant Biol. 13:166. 10.1186/1471-2229-13-166 24148786 PMC3854093

[B119] Potter D. (2012). Basic information on the stone fruit crops, in Genetics, Genomics and Breeding of Stone Fruits, eds Kole C. Abbott A. G. (New York, NY: CRC Press), 1–21.

[B120] Rahim M. A. Busatto N. Trainotti L. (2014). Regulation of anthocyanin biosynthesis in peach fruits. Planta 240, 913–929. 10.1007/s00425-014-2078-2 24827911

[B121] Ravaglia D. Espley R. V. Henry-Kirk R. A. Andreotti C. Ziosi V. Hellens R. P.. (2013). Transcriptional regulation of flavonoid biosynthesis in nectarine (*Prunus persica*) by a set of R2R3 MYB transcription factors. BMC Plant Biol. 13:68. 10.1186/1471-2229-13-68 23617716 PMC3648406

[B122] Raventós D. Skriver K. Schlein M. Karnahl K. Rogers S. W. Rogers J. C.. (1998). HRT, a novel zinc finger, transcriptional repressor from barley. J. Biol. Chem. 273, 23313–23320. 10.1074/jbc.273.36.23313 9722564

[B123] Richards D. E. Peng J. Harberd N. P. (2000). Plant GRAS and metazoan STATs: one family? Bioessays 22, 573–577. 10.1002/(SICI)1521-1878(200006)22:6<573::AID-BIES10>3.0.CO;2-H 10842311

[B124] Riechmann J. L. (2006). Transcription factors of *Arabidopsis* and rice: a genomic prespective. Annu. Plant Rev. 29, 28–53. 10.1002/9780470988886.ch2

[B125] Riechmann J. L. Meyerowitz E. M. (1998). The AP2/EREBP family of plant transcription factors. Biol. Chem. 379, 633–646. 9687012 10.1515/bchm.1998.379.6.633

[B126] Rios G. Tadeo F. R. Leida C. Badenes M. L. (2013). Prediction of components of the sporopollenin synthesis pathway in peach by genomic and expression analyses. BMC Genomics 14:40. 10.1186/1471-2164-14-40 23331975 PMC3556096

[B127] Rizhsky L. Liang H. Mittler R. (2002). The combined effect of drought stress and heat shock on gene expression in tobacco. Plant Physiol. 130, 1143–1151. 10.1104/pp.006858 12427981 PMC166635

[B128] Roberti M. Polosa P. L. Bruni F. Manzari C. Deceglie S. Gadaleta M. N.. (2009). The MTERF family proteins: mitochondrial transcription regulators and beyond. Biochim. Biophys. Acta 1787, 303–3011. 10.1016/j.bbabio.2009.01.013 19366610

[B129] Rodamilans B. San León D. Mühlberger L. Candresse T. Neumüller M. Oliveros J. C.. (2014). Transcriptomic analysis of *Prunus domestica* undergoing hypersensitive response to plum pox virus infection. PLoS ONE 9:e100477. 10.1371/journal.pone.0100477 24959894 PMC4069073

[B130] Romeu J. F. Monforte A. J. Sánchez G. Granell A. García-Brunton J. Badenes M. L.. (2014). Quantitative trait loci affecting reproductive phenology in peach. BMC Plant Biol. 14:52. 10.1186/1471-2229-14-52 24559033 PMC3941940

[B131] Rubio M. Rodríguez-Moreno L. Ballester A. R. Castro de Moura M. Bonghi C. Candresse T.. (2015). Analysis of gene expression changes in peach leaves in response to *Plum pox virus* infection using RNA-Seq. Mol. Plant Pathol. 16, 164–176. 10.1111/mpp.12169 24989162 PMC6638525

[B132] Ruiz K. B. Trainotti L. Bonghi C. Ziosi V. Costa G. Torrigiani P. (2013). Early methyl jasmonate application to peach delays fruit/seed development by altering the expression of multiple hormone-related genes. J. Plant Growth Regul. 32, 852–864. 10.1007/s00344-013-9351-7

[B133] Rushton P. J. Somssich I. E. Ringler P. Shen Q. J. (2010). WRKY transcription factors. Trends Plant Sci. 15, 247–258. 10.1016/j.tplants.2010.02.006 20304701

[B134] Sakuma Y. Liu Q. Dubouzet J. G. Abe H. Shinozaki K. Yamaguchi-Shinozaki K. (2002). DNA-binding specificity of the ERF/AP2 domain of Arabidopsis DREBs, transcription factors involved in dehydration- and cold-inducible gene expression. Biochem. Biophys. Res. Commun. 290, 998–1009. 10.1006/bbrc.2001.6299 11798174

[B135] Salazar J. A. Rubio M. Ruiz D. Tartarini S. Martínez-Gómez P. Dondini L. (2015). SNP development for genetic diversity analysis in apricot. Tree Genet. Genomes 11, 15 10.1007/s11295-015-0845-2

[B136] Salazar J. A. Ruiz D. Campoy J. A. Sánchez-Pérez R. Crisosto C. H. Martínez-García P. J. (2014). Quantitative Trait Loci (QTL) and Mendelian Trait Loci (MTL) analysis in *Prunus*: a breeding perspective and beyond. Plant Mol. Biol. Rep. 32, 1–18. 10.1007/s11105-013-0643-7

[B137] Salinas M. Xing S. Höhmann S. Berndtgen R. Huijser P. (2012). Genomic organization, phylogenetic comparison and differential expression of the SBP-box family of transcription factors in tomato. Planta 235, 1171–1184. 10.1007/s00425-011-1565-y 22160465

[B138] Sánchez G. Venegas-Calerón M. Salas J. J. Monforte A. Badenes M. L. Granell A. (2013). An integrative “omics” approach identifies new candidate genes to impact aroma volatiles in peach fruit. BMC Genomics 14:343. 10.1186/1471-2164-14-343 23701715 PMC3685534

[B139] Sánchez-Pérez R. Del Cueto J. Dicenta F. Martínez-Gómez P. (2014). Recent advancements to study flowering time in almond and other *Prunus* species. Front. Plant Sci. 5:334. 10.3389/fpls.2014.00334 25071812 PMC4093751

[B140] Sánchez-Pérez R. Dicenta F. Martínez-Gómez P. (2012). Inheritance of chilling and heat requirements for flowering in almond and QTL analysis. Tree Genet. Genomes 8, 379–389. 10.1007/s11295-011-0448-5

[B141] Santi L. Wang Y. Stile M. R. Berendzen K. Wanke D. Roig C.. (2003). The GA octodinucleotide repeat binding factor BBR participates in the transcriptional regulation of the homeobox gene Bkn3. Plant J. 34, 813–826. 10.1046/j.1365-313X.2003.01767.x 12795701

[B142] Santos A. M. Oliver M. J. Sanchez A. M. Oliveira M. M. (2012). Expression of almond Knotted1 homologue (PdKn1) anticipates adventitious shoot initiation. In Vitro Cell. Dev. Biol. Plant 48, 4–49. 10.1007/s11627-011-9388-x

[B143] Schauser L. Roussis A. Stiller J. Stougaard J. (1999). A plant regulator controlling development of symbiotic root nodules. Nature 402, 191–195. 10.1038/46058 10647012

[B144] Schiefthaler U. Balasubramanian S. Sieber P. Chevalier D. Wisman E. Schneitz K. (1999). Molecular analysis of NOZZLE, a gene involved in pattern formation and early sporogenesis during sex organ development in *Arabidopsis thaliana*. Proc. Natl. Acad. Sci. U.S.A. 96, 11664–11669. 10.1073/pnas.96.20.11664 10500234 PMC18091

[B145] Shen X. Zhao K. Liu L. Zhang K. Yuan H. Liao X.. (2014). A role for PacMYBA in ABA-regulated anthocyanin biosynthesis in red-colored sweet cherry cv. Hong Deng (Prunus avium L.). Plant Cell Physiol. 55, 862–880. 10.1093/pcp/pcu013 24443499

[B146] Sherif S. El-Sharkawy I. Paliyath G. Jayasankar S. (2013). PpERF3b, a transcriptional repressor from peach, contributes to disease susceptibility and side branching in EAR-dependent and -independent fashions. Plant Cell Rep. 32, 1111–1124. 10.1007/s00299-013-1405-6 23515898

[B147] Sherif S. Paliyath G. Jayasankar S. (2012). Molecular characterization of peach PR genes and their induction kinetics in response to bacterial infection and signaling molecules. Plant Cell Rep. 31, 697–711. 10.1007/s00299-011-1188-6 22101723

[B148] Shigyo M. Hasebe M. Ito M. (2006). Molecular evolution of the AP2 subfamily. Gene 366, 256–265. 10.1016/j.gene.2005.08.009 16388920

[B149] Shore P. Sharrocks A. D. (1995). The MADS-BOX family of transcription factors. Eur. J. Biochem. 229, 1–13. 10.1111/j.1432-1033.1995.tb20430.x 7744019

[B150] Shulaev V. Korban S. S. Sosinski B. Abbott A. G. Aldwinckle H. S. Folta K. M.. (2008). Multiple models for *Rosaceae* genomics. Plant Physiol. 147, 985–1003. 10.1104/pp.107.115618 18487361 PMC2442536

[B151] Siefers N. Dang K. K. Kumimoto R. W. Bynum W. E. Tayrose G. Holt B. F. (2009). Tissue-specific expression patterns of Arabidopsis NF-Y transcription factors suggest potential for extensive combinatorial complexity. Plant Physiol. 149, 625–641. 10.1104/pp.108.130591 19019982 PMC2633833

[B152] Silva C. García-Mas J. Sánchez A. M. Arús P. Oliveira M. M. (2005). Looking into flowering time in almond (*Prunus dulcis* (Mill) D.A. Webb): the candidate gene approach. Theor. Appl. Genet. 110, 959–968. 10.1007/s00122-004-1918-z 15700145

[B153] Smalle J. Kurepa J. Haegman M. Gielen J. Van Montagu M. Van Der Straeten D. (1998). The trihelix DNA-binding motif in higher plants is not restricted to the transcription factors GT-1 and GT-2. Proc. Natl. Acad. Sci. U.S.A. 95, 3318–3322. 10.1073/pnas.95.6.3318 9501260 PMC19739

[B154] Socquet-Juglard D. Kamber T. Pothier J. F. Christen D. Gessler G. Duffy B.. (2013). Comparative RNA-Seq analysis of early-infected peach leaves by the invasive phytopathogen *Xanthomonas arboricola pv*. Pruni. PLoS ONE 8:e54196. 10.1371/journal.pone.0054196 23342103 PMC3544827

[B155] Solano R. Stepanova A. Chao Q. Ecker J. R. (1998). Nuclear events in ethylene signaling: a transcriptional cascade mediated by ETHYLENE-INSENSITIVE3 and ETHYLENE-RESPONSE-FACTOR1. Genes Dev. 12, 3703–3714. 10.1101/gad.12.23.3703 9851977 PMC317251

[B156] Sooriyapathirana S. S. Khan A. Sebolt A. M. Wang D. C. Bushakra J. M. Lin-Wang K. (2010). QTL analysis and candidate gene mapping for skin and flesh color in sweet cherry fruit (*Prunus avium* L.). Tree Genet. Genomes 6, 821–832. 10.1007/s11295-010-0294-x

[B157] Soto A. Ruiz K. B. Ziosi V. Costa G. Torrigiani P. (2012). Ethylene and auxin biosynthesis and signaling are impaired by methyl jasmonate leading to a transient slowing down of ripening in peach fruit. J. Plant Physiol. 169, 1858–1865. 10.1016/j.jplph.2012.07.007 22884412

[B158] Souer E. van Houwelingen A. Kloos D. Mol J. Koes R. (1996). The No Apical Meristem gene of petunia is required for pattern formation in embryos and flowers and is expressed as meristem and primordia boundaries. Cell 85, 159–170. 10.1016/S0092-8674(00)81093-4 8612269

[B159] Stephen J. R. Dent K. C. Finch-Savage W. E. (2004). Molecular responses of *Prunus avium* (wild cherry) embryonic axes to temperatures affecting dormancy. New Phytol. 161, 401–413. 10.1046/j.1469-8137.2003.00927.x 33873512

[B160] Stracke R. Werber M. Weisshaar B. (2001). The R2R3-MYB geen family in *Arabidopsis thaliana*. Curr. Opin. Plant Biol. 4, 447–456. 10.1016/S1369-5266(00)00199-0 11597504

[B161] Sunkar R. Li Y. F. Jagadeeswaran G. (2012). Functions of microRNAs in plant stress responses. Trends Plant Sci. 17, 196–203. 10.1016/j.tplants.2012.01.010 22365280

[B162] Suzuki M. Kao C. Y. McCarty D. R. (1997). The conserved B3 domain of VIPAROUS1 has a cooperative DNA binding activity. Plant Cell 9, 799–807. 10.1105/tpc.9.5.799 9165754 PMC156957

[B163] Tadiello A. Pavanello A. Zanin D. Caporali E. Colombo L. Rotino G. L.. (2009). A PLENA-like gene of peach is involved in carpel formation and subsequent transformation into a fleshy fruit. J. Exp. Bot. 60, 651–661. 10.1093/jxb/ern313 19264761 PMC2651465

[B164] Takatsuji H. (1999). Zinc-finger proteins: the classical zinc finger emerges in contemporary plant science. Plant Mol. Biol. 39, 1073–1078. 10.1023/A:1006184519697 10380795

[B165] Tan K. Tegner J. Ravasi T. (2008). Integrated approaches to uncovering transcription regulatory networks in mammalian cells. Genomics 91, 219–231. 10.1016/j.ygeno.2007.11.005 18191937

[B166] Tani E. Polidoros A. N. Tsaftaris A. S. (2007). Characterization and expression analysis of FRUITFULL- and SHATTERPROOF-like genes from peach (*Prunus persica*) and their role in split-pit formation. Tree Physiol. 27, 649–659. 10.1093/treephys/27.5.649 17267356

[B167] Tani E. Tsaballa A. Stedel C. Kalloniati C. Papaefthimiou D. Polidoros A.. (2011). The study of a SPATULA-like bHLH transcription factor expressed during peach (*Prunus persica*) fruit development. Plant Physiol. Biochem. 49, 654–663. 10.1016/j.plaphy.2011.01.020 21324706

[B168] Teakle G. R. Manfield I. W. Graham J. F. Gilmartin P. M. (2002). *Arabidopsis thaliana* GATA factors: organization, expression and DNA-binding characteristics. Plant Mol. Biol. 50, 43–57. 10.1023/A:1016062325584 12139008

[B169] Testone G. Bruno L. Condello E. Chiappetta A. Bruno A. Mele G.. (2008). Peach [*Prunus persica* (L.) Batsch] KNOPE1, a class 1 KNOX orthologue to *Arabidopsis* BREVIPEDICELLUS/KNAT1, is misexpressed during hyperplasia of leaf curl disease. J. Exp. Bot. 59, 389–402. 10.1093/jxb/erm317 18250078

[B170] Testone G. Condello E. Verde I. Caboni E. Iannelli M. A. Bruno L.. (2009). The peach (*Prunus persica* [L.] Batsch) homeobox gene KNOPE3, which encodes a class 2 knotted-like transcription factor, is regulated during leaf development and triggered by sugars. Mol. Genet. Genomics 282, 47–64. 10.1007/s00438-009-0445-7 19333623

[B171] Testone G. Condello E. Verde I. Nicolodi C. Caboni E. Dettori M. T.. (2012). The peach (*Prunus persica* L. Batsch) genome harbours 10 KNOX genes, which are differentially expressed in stem development, and the class 1 KNOPE1 regulates elongation and lignification during primary growth. J. Exp. Bot. 63, 5417–5435. 10.1093/jxb/ers194 22888130 PMC3444263

[B172] Tittarelli A. Santiago M. Morales A. Meisel L. A. Silva H. (2009). Isolation and functional characterization of cold-regulated promoters, by digitally identifying peach fruit cold-induced genes from a large EST dataset. BMC Plant Biol. 9:121. 10.1186/1471-2229-9-121 19772651 PMC2754992

[B173] Trainin T. Bar-Ya'akov I. Holland D. (2013). ParSOC1, a MADS-BOX gene closely related to Arabidopsis AGL20/SOC1, is expressed in apricot leaves in a diurnal manner and is linked with chilling requirements for dormancy break. Tree Genet. Genomes 9, 753–766. 10.1007/s11295-012-0590-8

[B174] Trainotti L. Tadiello A. Casadoro G. (2007). The involvement of auxin in the ripening of climacteric fruits comes of age: the hormone plays a role of its own and has an intense interplay with ethylene in ripening peaches. J. Exp. Bot. 58, 3299–3308. 10.1093/jxb/erm178 17925301

[B175] Uematsu C. Katayama H. Makino I. Inagaki A. Arakawa O. Martin C. (2014). Peace, a MYB-like transcription factor, regulates petal pigmentation in flowering peach “Genpei” bearing variegated and fully pigmented flowers. J. Exp. Bot. 65, 1081–1094. 10.1093/jxb/ert456 24453228 PMC3935565

[B176] Ulmasov T. Hagen G. Guilfoyle T. J. (1997). ARF1, a transcription factor that binds to auxin response elements. Science 276, 1865–1868. 10.1126/science.276.5320.1865 9188533

[B177] Vendramin E. Pea G. Dondini L. Pacheco I. Dettori M. T. Gazza L.. (2014). A unique mutation in a MYB gene cosegregates with the nectarine phenotype in peach. PLoS ONE 9:e90574. 10.1371/journal.pone.0090574 24595269 PMC3940905

[B178] Verde I. Abbott A. G. Scalabrin S. Jung S. Shu S. Marroni F.. (2013). The high-quality draft of peach (*Prunus persica*) identifies unique patterns of genetic diversity, domestication and genome evolution. Nat. Genet. 45, 487–494. 10.1038/ng.2586 23525075

[B179] Verde I. Bassil N. Scalabrin S. Gilmore B. Lawley C. T. Gasic K.. (2012). Development and evaluation of a 9K SNP array for peach by internationally coordinated SNP detection and validation in breeding germplasm. PLoS ONE 7:e35668. 10.1371/journal.pone.0035668 22536421 PMC3334984

[B180] Verde I. Shu S. Jenkins J. Zuccolo A. Dettori M. T. Dardick C. (2015). The peach v2.0 release: an improved genome sequence for bridging the gap between genomics and breeding in *Prunus*, in Plant & Animal Genome XXIII Conference (San Diego, CA).

[B181] Vinson J. Su X. Zubik L. Bose P. (2001). Phenol antioxidant quantity and quality in foods: fruits. J. Agric. Food Chem. 49, 5315–5321. 10.1021/jf0009293 11714322

[B182] Vrebalov J. Pan I. L. Arroyo A. J. M. McQuinn R. Chung M. Poole M.. (2009). Fleshy fruit expansion and ripening are regulated by the tomato SHATTERPROOF gene TAGL1. Plant Cell 21, 3041–3062. 10.1105/tpc.109.066936 19880793 PMC2782289

[B183] Wang J. Zhang X. Yan G. Zhou Y. Zhang K. (2013). Over-expression of the PaAP1 gene from sweet cherry (*Prunus avium* L.) causes early flowering in *Arabidopsis thaliana*. J. Plant Physiol. 170, 315–320. 10.1016/j.jplph.2012.09.015 23206932

[B184] Wang X. L. Peng F. T. Yang L. Li M. J. Zhang S. S. (2012). Expression of genes involved in nitrate signaling and metabolism in peach roots in response to elevated levels of nitrate. Plant Mol. Biol. Rep. 30, 1450–1460. 10.1007/s11105-012-0462-2

[B185] Wang Y. Pijut P. M. (2013). Isolation and characterization of a TERMINAL FLOWER 1 homolog from *Prunus serotina* Ehrh. Tree Physiol. 33, 855–865. 10.1093/treephys/tpt051 23956129

[B186] Watson J. D. Gann A. Baker T. A. Levine M. Bell S. P. Harrison S. C. (2014). Molecular Biology of the Gene. New York, NY: Cold Spring Harbor; Cold Spring Harbor Laboratory Press.

[B187] Wells C. E. Vendramin E. Jiménez-Tarodo S. Verde I. Bielenberg D. G. (2015). A genome-wide analysis of MADS-BOX genes in peach [*Prunus persica* (L.) Batsch]. BMC Plant Biol. 15:41. 10.1186/s12870-015-0436-2 25848674 PMC4329201

[B188] Windhövel A. Hein I. Dabrowa R. Stockhaus J. (2001). Characterization of a novel class of plant homeodomain proteins that bind to the C4 phosphoenolpyruvate carboxylase gene of *Flaveria trinervia*. Plant Mol. Biol. 45, 201–214. 10.1023/A:1006450005648 11289511

[B189] Wisnieski M. Norelli J. Bassete C. Artlip T. Macarisin D. (2011). Ectopic expression of a novel peach (*Prunus persica*) CBF transcription factor in apple results in short-day induced dormancy and increased hardiness. Planta 233, 971–983. 10.1007/s00425-011-1358-3 21274560

[B190] Wu X. Shiroto Y. Kishitani S. Ito Y. Toriyama K. (2009). Enhanced heat and drought tolerance in transgenic rice seedlings overexpressing OsWRKY11 under the control of HSP101 promoter. Plant Cell Rep. 28, 21–30. 10.1007/s00299-008-0614-x 18818929

[B191] Xia R. Zhu H. An Y. Beers E. P. Liu Z. (2012). Apple miRNAs and tasiRNAs with novel regulatory networks. Genome Biol. 13:R47. 10.1186/gb-2012-13-6-r47 22704043 PMC3446319

[B192] Xu Y. Zhang L. Xie H. Zhang Y. Q. Oliveira M. M. Ma R. C. (2008). Expression analysis and genetic mapping of three SEPALLATA-like genes from peach (*Prunus persica* (L.) Batsch). Tree Genet. Genomes 4, 693–703. 10.1007/s11295-008-0143-3

[B193] Yamada Y. Wang H. Y. Fukuzawa M. Barton G. J. Williams J. G. (2008). A new family of transcription factors. Development 135, 3093–3101. 10.1242/dev.026377 18701541 PMC3586674

[B194] Yamane H. Kashiwa Y. Ooka T. Tao R. Yonemori K. (2008). Suppression subtractive hybridization and differential screening reveals endodormancy-associated expression of an SVP/AGL24-type MADS-BOX gene in lateral vegetative buds of japanese apricot. J. Amer. Soc. Hort. Sci. 133, 708–716.

[B195] Yamane H. Ooka T. Jotatsu H. Sasaki R. Tao R. (2011). Expression analysis of *PpDAM5* and *PpDAM6* during flower bud development in peach (*Prunus persica*). Sci. Hort. 129, 844–848. 10.1016/j.scienta.2011.05.01321378115

[B196] Yanagisawa S. (1997). Dof DNA-binding domains of plant transcription factors contribute to multiple protein-protein interactions. Eur. J. Biochem. 250, 403–410. 10.1111/j.1432-1033.1997.0403a.x 9428691

[B197] Yin Y. Vafeados D. Tao Y. Yoshida S. Asami T. Chory J. (2005). A new class of transcription factors mediates brassinosteroid-regulated gene expression in Arabidopsis. Cell 120, 249–259. 10.1016/j.cell.2004.11.044 15680330

[B198] Yu J. K. Paik H. Choi J. P. Han J. H. Choe J. K. Hur C. G. (2010). Functional domain marker (FDM): an *in silico* demonstration in *Solanaceae* using simple sequence repeats (SSRs). Plant Mol. Biol. Rep. 28, 352–356. 10.1007/s11105-009-0154-8

[B199] Zhang J. Yang W. R. Cheng T. R. Pan H. T. Zhang Q. X. (2013). Functional and evolutionary analysis of two CBF genes in *Prunus mume*. Can. J. Plant Sci. 93, 455–464. 10.4141/cjps2012-193

[B200] Zhang L. Xu Y. Ma R. C. (2008). Molecular cloning, identification, and chromosomal localization of two MADS-BOX box genes in peach (*Prunus persica*). J. Genet. Genomics 35, 365–372. 10.1016/S1673-8527(08)60053-3 18571125

[B201] Zhang Q. Chen W. Sun L. Zhao F. Huang B. Yang W.. (2012). The genome of *Prunus mume*. Nat. Commun. 3, 1318. 10.1038/ncomms2290 23271652 PMC3535359

[B202] Zhebentyayeva T. N. Fan S. Chandra A. Bielenberg D. G. Reighard G. L. Okie W. R. (2014). Dissection of chilling requirement and bloom date QTLs in peach using a whole genome sequencing of sibling trees from an F2 mapping population. Tree Genet. Genomes 10, 35–51. 10.1007/s11295-013-0660-6

[B203] Zheng N. Fraenkel E. Pabo C. O. Pavletich N. P. (1999). Structural basis of DNA recognition by the heterodimeric cell cycle transcription factor E2F-DP. Genes Dev. 13, 666–674. 10.1101/gad.13.6.666 10090723 PMC316551

[B204] Zhong W. Gao Z. Zhuang W. Shi T. Zhang Z. Ni Z. (2013). Genome-wide expression profiles of seasonal bud dormancy at four critical stages in Japanese apricot. Plant Mol. Biol. 83, 247–264. 10.1007/s11103-013-0086-4 23756818

[B205] Zhou D. X. Bisanz-Seyer C. Mache R. (1995). Molecular cloning of a small DNA binding protein with specificity for a tissue-specific negative element within the rps1 promoter. Nucleic Acids Res. 23, 1165–1169. 10.1093/nar/23.7.1165 7739894 PMC306826

[B206] Zhou H. Lin-Wang K. Wang H. Gu C. Dare A. P. Espley R. V.. (2015). Molecular genetics of blood-fleshed peach reveals activation of anthocyanin biosynthesis by NAC transcription factors. Plant J. 82, 105–121. 10.1111/tpj.12792 25688923

[B207] Zhou Y. Guo D. Li J. Cheng J. Zhou H. Gu C. (2013). Coordinated regulation of anthocyanin biosynthesis through photorespiration and temperature in peach (*Prunus persica* f. atropurpurea). Tree Genet. Genomes 9, 265–278. 10.1007/s11295-012-0552-1

[B208] Zhou Y. Zhou H. Lin-Wand K. Vimolmangkang S. Esplet R. V. Wang L.. (2014). Transcriptome analysis and transient transformation suggest an ancient duplicated MYB transcription factor as candidate gene for leaf red coloration in peach. BMC Plant Biol. 14:375. 10.1186/s12870-014-0388-y 25551393 PMC4302523

[B209] Zhu H. Xia R. Zhao B. Y. An Y. Q. Dardick C. D. Callahan A. M.. (2012). Unique expression, processing regulation, and regulatory network of peach (*Prunus persica*) miRNAs. BMC Plant Biol. 12:149. 10.1186/1471-2229-12-149 22909020 PMC3542160

[B210] Ziliotto F. Begheldo M. Rasori A. Bonghi C. Tonutti P. (2008). Transcriptome profiling of ripening nectarine (*Prunus persica* L. Batsch) fruit treated with 1-MCP. J. Exp. Bot. 59, 2781–2791. 10.1093/jxb/ern136 18515268 PMC2486471

[B211a] Ziosi V. Bregoli A. M. Fregola F. Costa G. Torrigiani P. (2009). Jasmonate-induced ripening delay is associated with up-regulation of polyamine levels in peach fruit. J. Plant Physiol. 166, 938–946. 10.1016/j.jplph.2008.11.014 19185952

[B211] Ziosi V. Noferini M. Fiori G. Tadiello A. Trainotti L. Casadoro G. (2008). A new index based on vis spectroscopy to characterize the progression of ripening in peach fruit. Postharvest Biol. Technol. 49, 319–329. 10.1016/j.postharvbio.2008.01.017

[B212] Zouine M. Fu Y. Chateigner-Boutin A.-L. Mila I. Frasse P. Wang H.. (2014). Characterization of the tomato ARF gene family uncovers a multi-levels post-transcriptional regulation including alternative splicing. PLoS ONE 9:e84203. 10.1371/journal.pone.0084203 24427281 PMC3888382

[B213] Zuriaga E. Soriano J. M. Zhebentyayeva T. Romero C. Dardick C. Cañizares J.. (2013). Genomic analysis reveals MATH gene(s) as candidate(s) for Plum pox virus (PPV) resistance in apricot (*Prunus armeniaca* L.). Mol. Plant Pathol. 14, 663–677. 10.1111/mpp.12037 23672686 PMC6638718

